# Genetic Diversity and Inter‐Specific Phylogeny of Three Sympatric Cetacean Species (*Stenella* spp.) in Thai Territorial Waters Based on Mitochondrial and Nuclear DNA Markers

**DOI:** 10.1002/ece3.72322

**Published:** 2025-10-12

**Authors:** Promporn Piboon, Janine L. Brown, Patcharaporn Kaewmong, Kongkiat Kittiwattanawong, Sarisa Klinhom, Toshiaki Yamamoto, Korakot Nganvongpanit

**Affiliations:** ^1^ The School of Veterinary Medicine, Faculty of Veterinary Medicine Chiang Mai University Chiang Mai Thailand; ^2^ Smithsonian Conservation Biology Institute Center for Species Survival Front Royal Virginia USA; ^3^ Phuket Marine Biological Center Phuket Thailand; ^4^ Department of Marine and Coastal Resources Ratthaprasasanabhakti Building (Building B) The Government Complex Bangkok Thailand; ^5^ Department of Veterinary Nursing and Technology Nippon Veterinary and Life Science University Musashino Tokyo Japan

**Keywords:** control region, Marine mammals, microsatellite markers, pantropical spotted dolphin, spinner dolphin, striped dolphin

## Abstract

Three *Stenella* species, *S. attenuata* , *S. coeruleoalba* , and *S. longirostris* , are widely distributed throughout the Indo‐Pacific region, but genetic information on each population remains scarce. This gap is particularly notable in Thai waters, where these species co‐occur in overlapping oceanic habitats. In contrast, previous studies in the Atlantic have revealed moderate to high mitochondrial genetic diversity and signs of ongoing hybridization among *Stenella* species. These findings raise the question of whether similar genetic patterns exist in other regions. To address this, we investigated genetic diversity, population structure, potential hybridization, and historical demographic change among three *Stenella* dolphin species in Thai waters. We analyzed 113 stranded specimens using 20 microsatellite loci and partial mitochondrial DNA (mtDNA) control region sequences. Results revealed high genetic diversity across all species, with expected heterozygosity highest in *S. attenuata* (*He* = 0.689), followed by *S. longirostris* (*He* = 0.651), and then *S. coeruleoalba* (*He* = 0.607). By contrast, *S. longirostris* exhibits the highest mtDNA diversity (haplotype diversity (*H*) = 0.988, nucleotide diversity (*π*) = 0.0194), followed by *S. coeruleoalba* (*H* = 0.986, *π* = 0.0173), and *S. attenuata* (*H* = 0.768, *π* = 0.0125). No population structure was detected within individual species by Bayesian clustering methods. However, analysis of the combined dataset for all three species suggests the presence of three genetic clusters (*K* = 3), with evidence of admixture across species but no shared mtDNA haplotypes, indicative of male‐mediated gene flow. Unique haplotypes were also found in all three species inhabiting Thai waters. Differing demographic histories likely reflect species‐specific responses to past environmental changes and recent anthropogenic impacts. These findings provide critical genetic insights for monitoring and informing conservation strategies in Thailand and surrounding regions.

## Graphical abstract

This study investigated the genetic diversity, population structure, and hybridization among three Stenella dolphin species ( *S. longirostris* , *S. attenuata* , *S. coeruleoalba* ) in Thailand's Andaman Sea using microsatellite loci and mitochondrial DNA from 113 stranded specimens. High genetic diversity was observed in all species, with potential hybridization indicated by Bayesian clustering despite no shared mtDNA haplotypes across species. The findings highlight important genetic insights for monitoring and conserving these dolphin populations in Thai and adjacent waters.

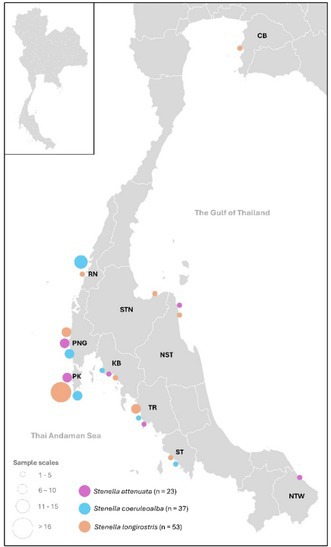

## 1 Introduction

Three dolphin species in the genus *Stenella*, the spotted dolphin ( *S. attenuata* ), the striped dolphin ( *S. coeruleoalba* ), and the spinner dolphin ( *S. longirostris* ), exhibit a pantropical distribution in all major oceans. All are currently listed as Least Concern on the IUCN Red List (Braulik and Reeves 2018; Kiszka and Braulik 2018; Braulik 2019). Despite overlapping distributions in Thai oceanic waters, particularly in the Andaman Sea (Central Database System and Data Standard for Marine and Coastal Resources 2013a), the genetic diversity, population structure, and potential interspecific interactions among these three species remain poorly understood. Existing knowledge is largely derived from stranding records, offering limited insight into population connectivity, gene flow, or demographic history. In contrast, studies from the Atlantic Ocean have revealed moderate to high mitochondrial diversity and evidence of interspecific gene flow among *Stenella* species, raising questions about whether similar patterns occur in Southeast Asia. Addressing this knowledge gap is essential for evaluating conservation status and informing regional management strategies.

The spotted dolphin is typically found in offshore and oceanic waters (Kiszka and Braulik 2018) and is widely distributed between 40° N and 40° S (Jefferson et al. 2015). Population estimates vary by region, with 15,917 individuals (CV = 40%) in the Hawaiian Archipelago, 50,880 individuals (CV = 27%) in the northern Gulf of Mexico, 3333 individuals (CV = 91%) along the east coast of North America, and 278,155 individuals (CV = 59%) in the Eastern Tropical Pacific, where it is most abundant (Kiszka and Braulik 2018). A distinct geographic form, *S. attenuata graffmani* , has been described and is found in coastal waters of the Eastern Tropical Pacific (Kiszka and Braulik 2018). The offshore pantropical spotted dolphin is the primary species affected by tuna purse‐seine fisheries in the Eastern Tropical Pacific. Additionally, it is threatened by drive fisheries, gillnets, and other fishing equipment in areas such as Japan, the Philippines, Sri Lanka, and the Solomon Islands.

The striped dolphin also has a wide distribution range in tropical to warm‐temperate ocean waters, but extends to higher latitudes than other *Stenella* species, up to 50° N and 40° S (Jefferson et al. 2015). This species is typically found outside the continental shelf, most often over the continental slope (Archer II 2018). It is the second‐most abundant species sighted in the western tropical Indian Ocean, with an estimated population of 570,000 (CV = 19%) in the western North Pacific based on data from 1983 to 1991 (Miyashita 1993). However, up‐to‐date population estimates are lacking, including for Thai waters. The species faces significant threats from human activities, being heavily impacted by harpoon and drive fisheries in Japan (Jefferson et al. 2015).

The spinner dolphin has a wide distribution range across all oceanic waters, ranging from 40° N to 40° S, but is absent from the Mediterranean Sea (Braulik and Reeves 2018). Four subspecies have been documented: Gray's spinner dolphin ( *S. longirostris longirostris* ), the most common and widely distributed subspecies in the tropical Atlantic, Indian, and western Pacific Oceans, including in the Thai Andaman Sea and the Gulf of Thailand; Eastern spinner dolphin ( *S. longirostris orientalis* ), found only in the waters of the Eastern Pacific Ocean and mostly in offshore waters; Central American spinner dolphin ( *S. longirostris centroamericana* ), distributed only in coastal waters of the eastern Pacific Ocean in Central America; and dwarf spinner dolphin ( *S. longirostris roseiventris* ), a small form distributed in shallow waters in the Gulf of Thailand, Indonesia, and southern China (Braulik and Reeves 2018). In the eastern Pacific Ocean, this species was heavily impacted by tuna purse‐seine fisheries, with more than 4 million killed between 1960 and 1972, potentially reducing the population in that area by 65% (Braulik and Reeves 2018; Wade et al. 2007). In the Gulf of Thailand, dwarf spinners have been caught in shrimp trawls (Perrin et al. 1999); however, information on the species in the Thai Andaman Sea is scarce.

Previous research in the southwest and northeast Atlantic Oceans has revealed moderate to high mitochondrial genetic diversity among five *Stenella* species (Haplotype diversity (*H*) = 0.833–1.000 and nucleotide diversity (*π*) = 0.006–0.015) and provided evidence of interspecific gene flow, likely facilitated by sympatric distributions, mixed‐species associations, and underlying genomic permeability (Faria et al. 2022). In several cases, individuals morphologically identified as one species were found to carry mitochondrial haplotypes of another, indicating potential introgressive hybridization and ongoing maternal gene flow between species (Faria et al. 2022). Such findings underscore the challenges in resolving phylogenetic relationships and taxonomy within this genus. In Thai waters, comprehensive data on species‐specific abundance, distribution, genetic structure, anthropogenic threats, and regional connectivity are still limited, hindering effective conservation planning (Braulik et al. 2018). To date, extensive research on cetaceans in Thai waters, including behavior, habitat use, and population genetics, has been conducted for only a few dolphin species: Irrawaddy dolphin (Hines et al. 2015; Jackson‐Ricketts et al. 2020; Ponnampalam et al. 2013; Junchompoo et al. 2014; Tongnunui et al. 2011; Niu et al. 2019; Jutapruet et al. 2017), Indo‐Pacific humpback dolphin (Zhao et al. 2021; Banlunara et al. 2019; Jaroensutasinee et al. 2010; Jutapruet, Huang, et al. 2015; Jutapruet, Kittiwattanawong, and Pradit 2015; Jutapruet et al. 2017; Dai et al. 2022), and Indo‐Pacific bottlenose dolphin (Prasitwiset et al. 2022), all of which are coastal species. For other species in Thai waters, information on population size and distribution is primarily based on visual surveys, opportunistic sightings, and stranding records. Examples include the Indo‐Pacific finless porpoise (Jutapruet et al. 2017), Bryde's whale (Thongsukdee et al. 2014; Cherdsukjai et al. 2015), spinner dolphin (Perrin et al. 1989), and killer whale ( *Orcinus orca* ) (Adulyanukosol et al. 2010). Given the evidence of mitochondrial diversity and introgressive hybridization among *Stenella* species in the Atlantic (Faria et al. 2022), it is plausible that similar patterns may occur in Southeast Asia, especially in regions where multiple species overlap. Yet, no genetic studies have examined this possibility in Thailand.

To address this gap, we investigated the genetic diversity, population structure, and potential for genetic admixture and interspecific gene flow among three *Stenella* species using both nuclear and mitochondrial markers. A total of 113 specimens, collected from strandings along the Thai coastline between 1998 and 2019, were analyzed using 20 microsatellite loci and partial sequences of the mitochondrial control region. This study provides the first comparative genetic baseline for these species in Thai waters, contributing essential information for evaluating their evolutionary history, regional connectivity, and conservation needs.

## 2 Materials and Methods

### 2.1 Sample Collection and DNA Extraction

**FIGURE 1:**
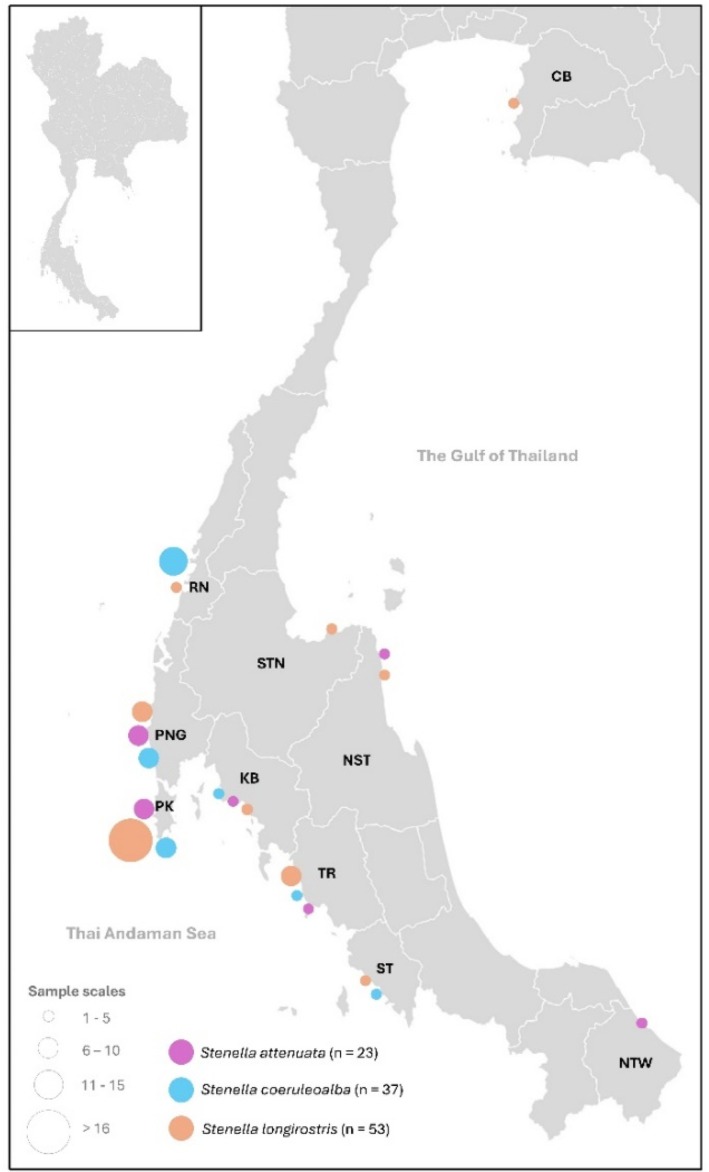
Sample locations of stranded *Stenella* spp. including *S. attenuata* (*n* = 23), *S. coeruleoalba* (*n* = 37), and *S. longirostris* (*n* = 53) in the Thai Andaman Sea and the Gulf of Thailand. Location abbreviations: CB, Chon Buri; STN, Surat Thani; NST, Nakhon Si Thammarat; NTW, Narathiwat; RN, Ranong; PNG, Pang Nga; PK, Phuket; KB, Krabi; TR, Trang; and ST, Satun.

Skin samples of three dolphin species [ *S. attenuata* (*n* = 23), *S. coeruleoalba* (*n* = 37), *S. longirostris* (*n* = 53)] from stranded animals along the Thai coast between 1998 and 2019 were provided by the Phuket Marine Biological Center (PMBC), Phuket, Thailand (Table S1). Most samples were collected from coastal areas of the Andaman Sea: Ranong (RN), Phang Nga (PNG), Phuket (PK), Krabi (KB), Trang (TR), and Satun (ST) (Figure 1). Only two samples of *S. attenuata* and three samples of *S. longirostris* were collected from the Gulf of Thailand (Figure 1). Species were identified based on morphological appearance at stranding sites by veterinarians of the PMBC. Samples were either frozen at −20°C or preserved in 95% ethanol until DNA extraction using DNA extraction kits according to the manufacturer's instructions (DNeasy Blood & Tissue Kit, QIAGEN, Germany) at the Laboratory of the Faculty of Veterinary Medicine, Chiang Mai University (Piboon, Kriengsakpichit, Poommouang, Buddhachat, et al. 2022; Piboon, Poommouang, Buddhachat, Kaewmong, et al. 2022; Chantra et al. 2023; Prasitwiset et al. 2022; Piboon, Poommouang, Buddhachat, Kampuansai, et al. 2022). This study was approved by the Animal Use Committee of the Faculty of Veterinary Medicine, Chiang Mai University, Thailand, in 2020 (S14/2563).

### 2.2 Microsatellite Genotyping

Twenty microsatellite loci were screened and amplified for genotyping in each species using polymerase chain reaction (PCR) (see Table S2 for all loci) (Escorza‐Trevino et al. 2005; Farro et al. 2008; Mirimin et al. 2006; Faria et al. 2022). PCR reactions were performed in 25 μL volumes using a PCR thermocycler (Bio‐Rad Laboratories Inc., CA, USA). Each reaction contained 0.2 mM dNTPs (Vivantis, Selangor Darul Ehsan, Malaysia), 0.2 μM of each primer (forward primer was 5′ M13 complementary tail to enable labeling with a fluorescent M13 primer, see Table S2), 1X PCR buffer (Vivantis, Selangor Darul Ehsan, Malaysia), 1 U *Taq* DNA polymerase (Vivantis, Selangor Darul Ehsan, Malaysia), and 10 ng/μL DNA template. Thermal cycling conditions were as follows: initial denaturation at 95°C for 5 min; followed by 40 cycles of 95°C for 30 s, annealing at the locus‐specific temperature for 45 s (Table S2), 72°C for 1 min; and the final extension step at 72°C for 10 min. The PCR products were separated by electrophoresis on a 2% agarose gel and visualized by UV light using GelMax 125 Imager (UVP, Cambridge, UK) to observe the amplified bands. Unsuccessful PCR reactions were repeated once or twice. Samples that failed to amplify were excluded from the analysis. Fragment analysis was performed using the Applied Biosystems 3730 XL‐96 Genetic Analyzer (Thermo Fisher Scientific, Waltham, MA, USA) at Ward Medic Ltd. (Bangkok, Thailand). The sizes of amplified microsatellite markers were determined manually using GENE MARKER version 2.6.2 (Hulce et al. 2011). Genotyping errors, such as null alleles, allele drop‐out, and stuttering, were assessed using Micro‐Checker version 2.2.3 (Van Oosterhout et al. 2004).

### 2.3 Mitochondrial DNA Sequencing

The mitochondrial DNA control regions were amplified using a pair of PCR primers: forward (5′‐CATATTACAACGGTCTTGTAAACC‐3′) and reverse (5′‐GTCATAAGTCCATCGAGATGTC‐3′). These primers amplify a 620 bp fragment spanning from the tRNA‐Pro gene to the middle of the control region (D‐loop) in many cetacean species (Piboon, Kriengsakpichit, Poommouang, Buddhachat, et al. 2022). PCR amplification was conducted in a 25 μL reaction volume containing 1X PCR buffer (Vivantis, Selangor Darul Ehsan, Malaysia), 0.2 mM dNTPs, 0.2 μM of both forward and reverse primers, 1 U *Taq* DNA polymerase (Vivantis, Selangor Darul Ehsan, Malaysia), and 0.2 ng/μL of the DNA sample, adjusted to 25 μL with deionized water. PCR cycling was performed as follows: initial denaturation at 95°C for 5 min; followed by 40 cycles of 95°C for 30 s, 50°C for 45 s, and 72°C for 1 min; with a final extension at 72°C for 10 min. The PCR products were visualized by electrophoresis in a 2% agarose gel before sequencing. Unsuccessful PCR reactions were repeated once or twice. Samples that failed to amplify were excluded from the analysis. The successful amplicons were then sequenced by ATGC Co. Ltd. (Pathum Thani, Thailand) using a 3730XL‐96 Genetic Analyzer (Thermo Fisher Scientific, MA, USA).

### 2.4 Data Analysis

#### 2.4.1 Microsatellite Data

Three microsatellite datasets were generated, one for each species. The program Genepop version 4.2 (Raymond 1995) was used to evaluate the deviations from the expected Hardy–Weinberg (HWE) genotypic frequencies using Fisher's exact test and 1000 permutations in the Markov chain. The polymorphic information content (PIC), cumulative probability of identity individuals (PID), and siblings (PID sibs) were estimated using CERVUS version 3.0.7 (Kalinowski et al. 2007). To estimate the level of polymorphism within each species dataset, allelic richness (*AR*), alleles per locus (*Na*), observed heterozygosity (*Ho*), expected heterozygosity (*He*), and inbreeding coefficient (*Fis*) were calculated using FSTAT version 2.9.3.2 (Goudet 2002). To assess population structure, two Bayesian clustering analyses were conducted using STRUCTURE version 2.3.4 (Pritchard et al. 2000). In the first analysis, each species dataset was analyzed independently. In the second analysis, all three species datasets were combined into a fourth, composite dataset to evaluate potential admixture among species using the same program. Both analyses were performed without the LOCPRIOR model (Pritchard et al. 2000). The structure analysis used a Markov Chain Monte Carlo (MCMC) approach with values of K ranging from 1 to 5 to infer population structure and estimate the number of clusters. Each run used the admixture population model with 300,000 iterations, a burn‐in of 50,000, and 10 independent replicates per K value. The optimal number of clusters (K) was determined using multiple criteria: the Pritchard method (Pr[X|K]) (Pritchard et al. 2000), the Evanno method (ΔK) (Evanno et al. 2005), and the Parsimony index (PI) (Wang 2019), through KFinder version 1.0.0.0 (Wang 2019). The outputs were then graphically visualized by DISTRUCT (Rosenberg 2004).

#### 2.4.2 Mitochondrial DNA

The mtDNA control region sequences were visually prepared and edited manually using MEGA‐X version 10.2.2 (Kumar et al. 2018). To ensure the accuracy of using morphology for species identification, all sequences were confirmed against the reference sequence in NCBI using Basic Local Alignment Search Tool (BLAST). The sequences were then grouped into three datasets according to species: *S. attenuata* (20 sequences), *S. coeruleoalba* (36 sequences), *S. longirostris* (42 sequences) (See accession number in Table S1). The sequences of each dataset were aligned using Clustal W in MEGA X version 10.2.2 (Kumar et al. 2018). Genetic diversity parameters, such as the number of haplotypes (*Nh*), number of polymorphic sites (*S*), haplotype diversity (*H*), and nucleotide diversity (*π*), were determined using DnaSP version 6.12.3 (Rozas et al. 2017). Median joining networks (MJNs) of each species dataset and the combined three‐species dataset were constructed to examine the relationships among maternal lineage haplotypes and the number of mutational steps. Analyses were performed using PopART version 1.7 (Leigh et al. 2015).

To examine maternal lineages on a global scale, sequences from Thai waters and publicly available sequences from other regions were obtained from the GenBank database and used to generate three datasets: *S. attenuata* (179 sequences) (Courbis et al. 2014; Escorza‐Trevino et al. 2005; Kingston et al. 2009; Oremus et al. 2015; Viricel and Rosel 2021, National Center for Biotechnology Information; Yusmalinda et al. 2017; Jayasankar et al. 2007, National Center for Biotechnology Information); *S. coeruleoalba* (167 sequences) (Garrigue et al. 2023; Floridia et al. 2013, National Center for Biotechnology Information; Galov et al. 2009; Kingston et al. 2009; Kitamura and Abe 2013, National Center for Biotechnology Information; Linguiti et al. 2021; Mace et al. 2006; Guang et al. 2002); *S. longirostris* (229 sequences) (Faria et al. 2022; Viricel et al. 2016; Martien et al. 2014; Oremus et al. 2007, 2015; Andrews et al. 2010, 2013) (See all accession numbers in Table S3). The MJNs of the global datasets were then constructed using PopART version 1.7 (Leigh et al. 2015). To evaluate taxonomic clustering, phylogenetic trees were constructed from the same sequence datasets using Bayesian inference implemented in MrBayes version 3.2.7 (Ronquist et al. 2011). The best‐fit evolutionary model (GTR + I + G) was selected using jModelTest version 2.1.10 (Posada 2008). Bayesian analysis was conducted using Markov Chain Monte Carlo (MCMC) sampling for 3,000,000 iterations, with trees sampled every 5000 iterations. Convergence was assessed based on an average standard deviation of split frequencies below 0.01. Final phylogenetic trees were visualized using iTOL version 6.1.1 (Letunic and Bork 2021).

To assess deviations from neutral evolution in each species in Thai waters, Tajima's D and Fu's Fs statistics were calculated for each species using DnaSP version 6.12.3 (Rozas et al. 2017). Statistical significance was evaluated using 10,000 bootstrap replicates. To examine the frequency distribution of pairwise nucleotide differences among sequences, mismatch distribution analyses were performed using DnaSP version 6.12.3 (Rozas et al. 2017). To investigate the frequency distribution of pairwise nucleotide differences among sequences, mismatch distribution analyses were also performed in DnaSP. Harpending's raggedness index (*r*) was calculated to quantify the smoothness of the mismatch distribution, with significance similarly assessed using 10,000 bootstrap replicates (Rozas et al. 2017; Oremus et al. 2007). Historical demographic trends were reconstructed using Bayesian Skyline Plots (BSPs) implemented in BEAST (Bouckaert et al. 2014), with convergence and effective sample sizes (ESS > 200) verified using Tracer version 1.7.2 (Rambaut et al. 2018). The best‐fitting substitution models were selected using jModelTest version 2.1.10 (Posada 2008): HKY for *S. attenuata* , HKY + I + G for *S. coeruleoalba* , and GTR + I for *S. longirostris* . Markov Chain Monte Carlo (MCMC) analyses were run for 10 million generations, sampling every 1000 steps, with a 10% burn‐in. We used a lognormal relaxed clock rate with a mutation rate for the control region of 6.3 × 10^−7^ substitution/site/year from *Lagenorhynchus obscurus* (Harlin et al. 2003).

## 3 Results

### 3.1 Estimate Genetic Diversity and Population Structure Based on Microsatellite Loci

A total of 14, 16, and 18 microsatellite loci were amplified for *S. attenuata* (*n* = 15/23), *S. coeruleoalba* (*n* = 34/37), and *S. longirostris* (*n* = 40/53), respectively (numbers in parentheses indicate numbers of samples successfully amplified out of the total number, Table S1). However, some loci were excluded from the genetic diversity and population structure analysis of individual species datasets due to incomplete amplification, the presence of null alleles, or because they were monomorphic (Table S2). Finally, four microsatellite datasets were generated: (1) 8 loci of *S. attenuata* (EV104‐HEX, Slo4‐FAM, Sco28‐ROX, Sco66‐TAMRA, Sl1‐25‐TAMRA, Sl8‐49‐FAM, Sl9‐69‐FAM, and 4EV1‐TAMRA), (2) 9 loci of *S. coeruleoalba* (EV104‐HEX, Slo4‐FAM, Slo9‐HEX, Slo15‐HEX, Sco11‐HEX, Sco28‐ROX, Sco65‐ROX, Sco66‐TAMRA, and Sl9‐69‐FAM), (3) 14 loci of *S. longirostris* (Ev37‐HEX, EV94‐ROX, EV104‐HEX, Slo4‐FAM, Slo9‐HEX, Slo15‐HEX, Sco11‐HEX, Sco28‐ROX, Sco55‐ROX, Sd8‐FAM, Sl1‐25‐TAMRA, Sl8‐49‐FAM, Sl9‐69‐FAM, and 4EV1‐TAMRA), and (4) 10 loci of a combined dataset of all three species (EV104‐HEX, Slo4‐FAM, Slo9‐HEX, Sco28‐ROX, Sco65‐ROX, Sl1‐25‐TAMRA, Sl8‐49‐FAM, Sl9‐69‐FAM, and 4EV1‐TAMRA). Monomorphic loci were retained for the population structure analysis of the combined dataset (Table S2). All microsatellite loci used in each dataset showed no significant deviation from the HWE (See the detail of each dataset in Tables S4–, S6).

The microsatellite panel demonstrated high levels of informativeness across the three *Stenella* species sampled in Thai waters. The average PIC values were 0.621 (±0.221) for *S. attenuata* , 0.570 (±0.235) for *S. coeruleoalba* , and 0.614 (±0.231) for *S. longirostris* , indicating that the markers were moderately to highly informative across species. Cumulative PID was lowest in *S. longirostris* at 5.899 × 10^−14^, compared to 6 × 10^−8^ for both *S. attenuata* and *S. coeruleoalba* . Similarly, cumulative PIDsibs was lowest in *S. longirostris* at 1.686 × 10^−5^, while *S. coeruleoalba* and *S. attenuata* showed slightly higher values of 1.554 × 10^−3^ and 1.648 × 10^−3^, respectively (Tables S4–, S6). For the combined microsatellite of three species, the cumulative PID was 4.01 × 10^−11^ and PIDsibs was 2.97 × 10^−4^, indicating strong power to differentiate individuals even among closely related ones. Microsatellite‐based genetic diversity estimates revealed interspecific differences among the *Stenella* species sampled in Thai waters (Table 1). Although *S. attenuata* exhibited the lowest number of alleles and allelic richness across eight loci, this pattern likely reflects its smaller sample size (*n* = 15) rather than true lower diversity. When using sample size–independent measures, *S. attenuata* actually exhibited the highest *He* (0.689 ± 0.218), indicating it may possess greater underlying genetic diversity than the other species. *S. coeruleoalba* had intermediate values, while *S. longirostris* , despite having the largest sample size (*n* = 40) and highest *Na* (8.93) and *AR* (8.47), had a lower *He* (0.651 ± 0.224) compared to *S. attenuata* . *Ho* was slightly lower than *He* in both *S. attenuata* and *S. longirostris* , suggesting mild heterozygote deficits (*Fis* = 0.051 and 0.084, respectively), whereas *S. coeruleoalba* showed a slight excess of heterozygotes (*Fis* = −0.013), possibly indicating random mating or outbreeding.

**TABLE 1:** Genetic diversity based on microsatellite loci of each *Stenella* spp. in Thai waters.

Species	*N*	*L*	*Na*	*AR*	*He* (SD)	*Ho* (SD)	*Fis* (SD)
*S. attenuata*	15/23	8	6.25	5.72	0.689 (0.218)	0.668 (0.242)	0.051 (0.247)
*S. coeruleoalba*	34/37	9	7.33	6.86	0.607 (0.241)	0.616 (0.253)	−0.013 (0.079)
*S. longirostris*	40/53	14	8.93	8.47	0.651 (0.224)	0.607 (0.237)	0.084 (0.080)

Abbreviations: *AR*, mean allelic richness; *Fis*, inbreeding coefficient; *He*, mean expected heterozygosity; *Ho*, mean observed heterozygosity; *L*, number of microsatellite loci; *N*, number of samples/all samples; *Na*, mean number of allele per locus; SD, standard deviation.

**FIGURE 2:**
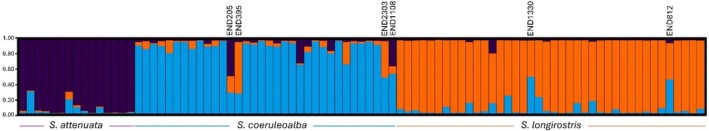
Bayesian clustering assignment based on 10 microsatellite loci of combined dataset of *Stenella* spp. The results consisted of three genetic clusters (the most likely K = 3) indicated by color (purple, blue and orange), which K is the assumed number of ancestral groups. Details of the species of the samples are given below the graphic. Each single vertical column represents an individual and the proportion of each color indicates the proportion of ancestry.

No population subdivision was detected within each species, as all individual datasets showed the most likely K value of 1 based on both the Pr[X|K] and PI methods (Table S7). However, when admixture among the three species was assessed using a combined dataset of 10 microsatellite loci in a Bayesian clustering analysis, the results supported a most likely K value of 3 across all criteria (Figure 2, Table S7). The population structure identified three genetic clusters, represented by purple, blue, and orange, corresponding to *S. attenuata, S. coeruleoalba*, and *S. longirostris*, respectively (Figure 2). Notably, six individuals showed ancestry patterns that deviated from their assigned species clusters. Four individuals identified as *S. coeruleoalba* (END205, END395, END2303, END1108) and two individuals identified as *S. longirostris* (END1330, END812) exhibited over 50% genetic contribution from other clusters (Figure 2). The individuals identified as *S. coeruleoalba* (END 205 and END1108) exhibited genetic proportions similar to *S. attenuata*, while END395 and END2303 were more like *S. longirostris* . Similarly, two individuals identified as *S. longirostris* (END 1330 and END812), showed similar genetic proportions to *S. coeruleoalba* (Figure 2).

### 3.2 Estimate Genetic Diversity, Maternal Lineage and Inter‐Specific Phylogeny Based on mtDNA Control Region

Sequencing of the mtDNA control region led to the identification of 7, 29, and 33 haplotypes for *S. attenuata* (*n* = 20/23), *S. coeruleoalba* (*n* = 36/37), and *S. longirostris* (*n* = 42/53), respectively (Table S2). The maximum number of polymorphic sites was found for *S. coeruleoalba* at 52 sites, while only 16 sites were detected in *S. attenuata* (Table 2). Haplotype diversity was high across all species, ranging from 0.768 to 0.988, and nucleotide diversity ranged from 0.0125 to 0.0194 (Table 2). While the number of haplotypes, polymorphic sites, and haplotype diversity generally increased with sample size, nucleotide diversity is considered the most unbiased measure of genetic diversity across unequal sample sizes. Based on π, *S. longirostris* exhibited the highest mtDNA diversity, followed by *S. coeruleoalba* and *S. attenuata* .

**TABLE 2:** Mitochondrial DNA control region diversity of each *Stenella* spp. in Thai waters.

Species	*N*	*Nh* (length)	*S*	*H* (SD)	*π* (SD)
*S. attenuata*	20/23	7 (547 bp)	16	0.768 (0.005)	0.0125 (0.001)
*S. coeruleoalba*	36/37	29 (484 bp)	52	0.986 (0.011)	0.0173 (0.002)
*S. longirostris*	42/53	33 (423 bp)	51	0.988 (0.008)	0.0194 (0.001)

Abbreviations: *π*, nucleotide diversity; *H*, haplotype diversity; *N*, number of sequences/all samples; *Nh*, number of haplotypes; *S*, number of polymorphic sites; SD, standard deviation.

**FIGURE 3:**
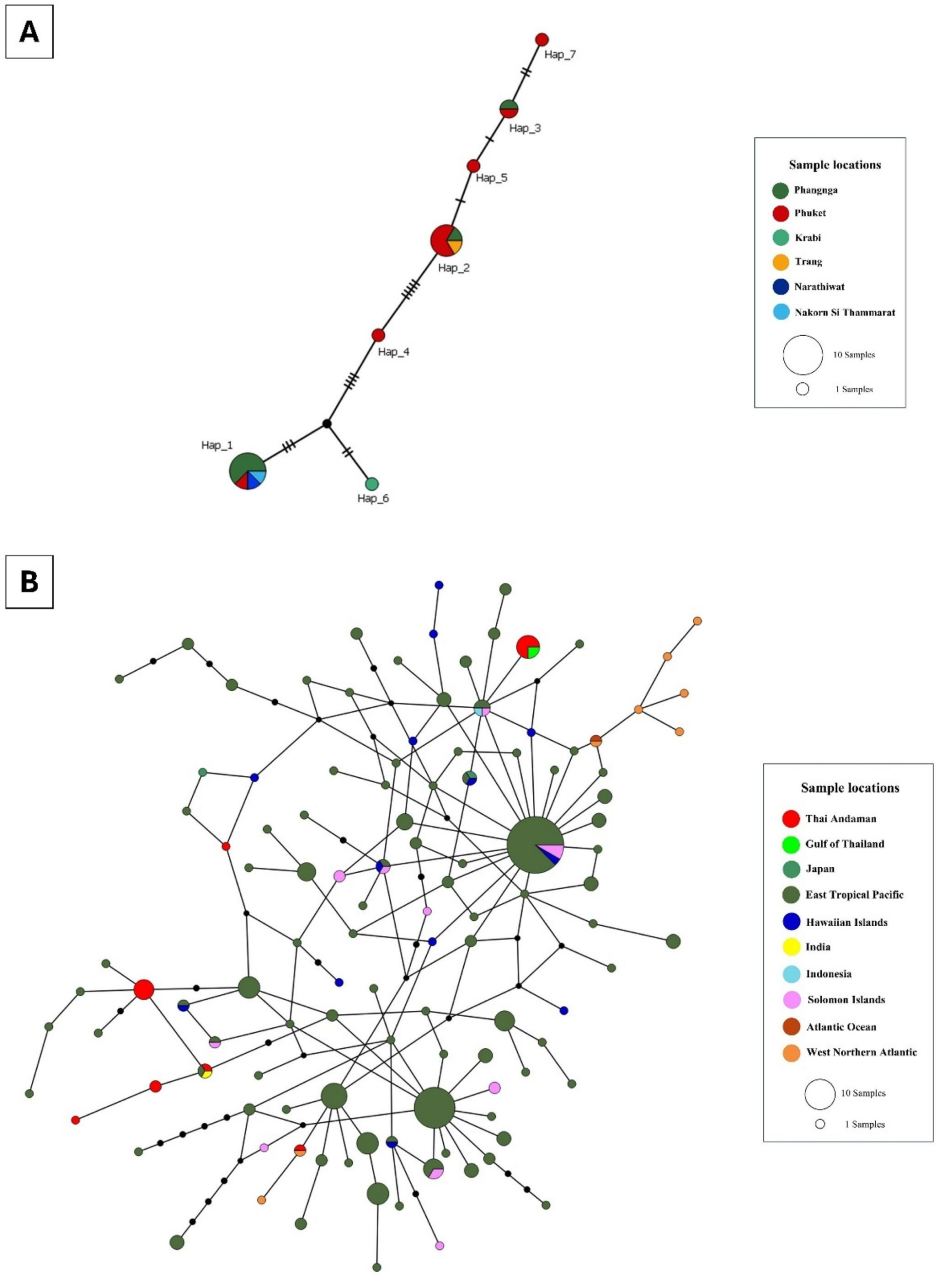
The median joining networks (MJNs) of *Stenella attenuata* . (A) The MJNs showed 7 haplotypes of *S. attenuata* from Thai Andaman Sea and the Gulf of Thailand generated by 547 bp of mtDNA control region sequences. Each haplotype is colored by sampling location. The small black circles represent inferred haplotypes (not sampled). The size of each circle is proportional to its haplotype frequency, while nucleotide substitutions are shown at the branches as small transverse bars. (B) The MJNs of 116 haplotypes of *S. attenuata* from 179 sequences worldwide (322 bp). Each haplotype is colored by sample location. The small black circles represent inferred haplotypes (not sampled). The size of each circle is proportional to its haplotype frequency, while nucleotide substitutions are shown as 1‐step edges.

**FIGURE 4:**
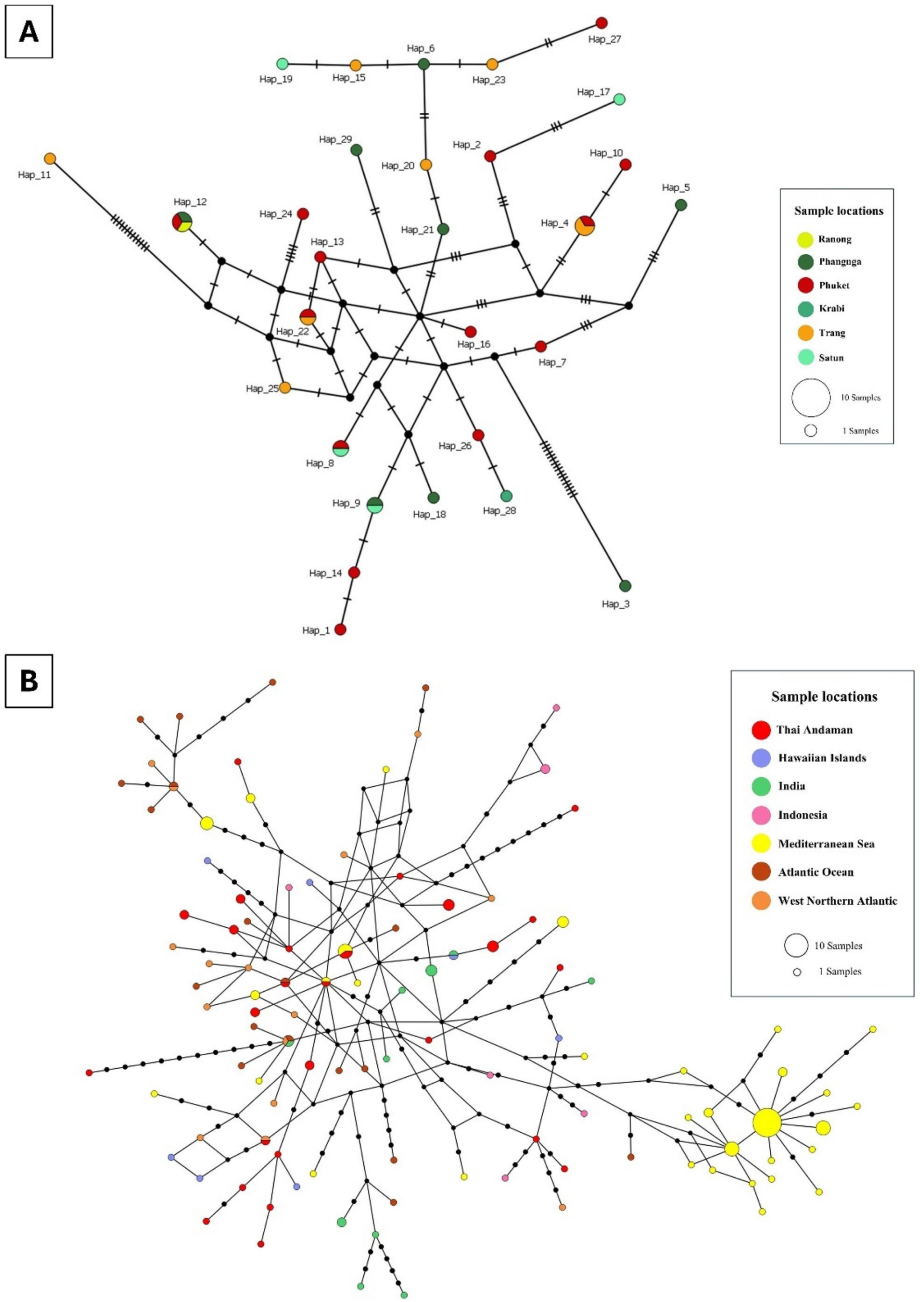
The median joining networks (MJNs) of *Stenella coeruleoalba* . (A) The MJNs showed 29 haplotypes of *S. coeruleoalba* from Thai Andaman Sea generated by 484 bp of mtDNA control region sequences. Each haplotype is colored by sampling location. The small black circles represent inferred haplotypes (not sampled). The size of each circle is proportional to its haplotype frequency, while nucleotide substitutions are shown at the branches as small transverse bars. (B) The MJNs of 107 haplotypes of *S. coeruleoalba* from 167 sequences worldwide (311 bp). Each haplotype is colored by sample location. The small black circles represent inferred haplotypes (not sampled). The size of each circle is proportional to its haplotype frequency, while nucleotide substitutions are shown as 1‐step edges.

**FIGURE 5:**
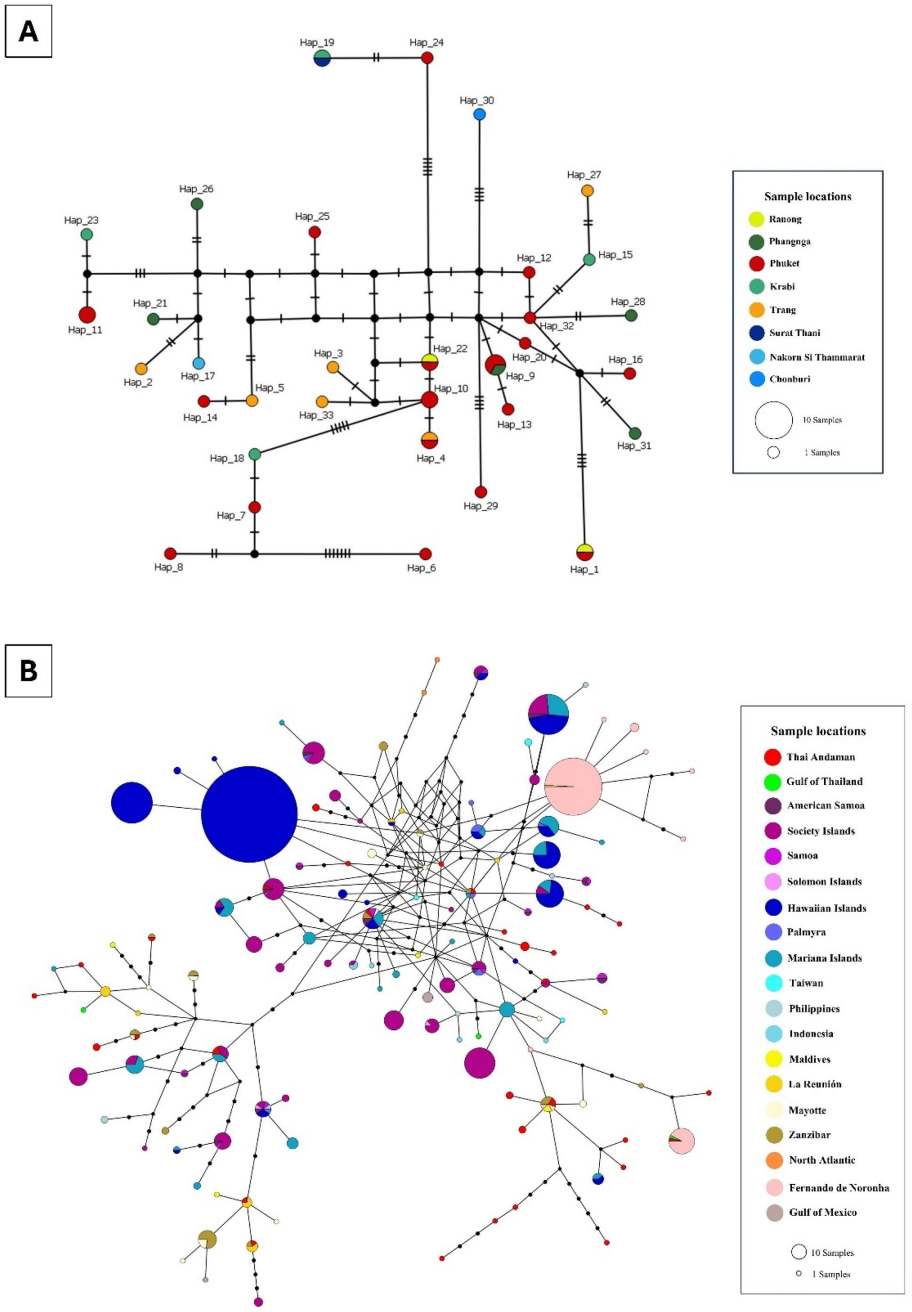
The median joining networks (MJNs) of *Stenella longirostris* . (A) The MJNs showed 33 haplotypes of *S. longirostris* from Thai Andaman Sea generated by 423 bp of mtDNA control region sequences. Each haplotype is colored by sampling location. The small black circles represent inferred haplotypes (not sampled). The size of each circle is proportional to its haplotype frequency, while nucleotide substitutions are shown at the branches as small transverse bars. (B) The MJNs of 129 haplotypes of *S. longirostris* from 229 sequences worldwide (374 bp). Each haplotype is colored by sample location. The small black circles represent inferred haplotypes (not sampled). The size of each circle is proportional to its haplotype frequency, while nucleotide substitutions are shown as 1‐step edges.

The MJNs of *S. attenuata, S. coeruleoalba*, and *S. longirostris* from Thai waters and worldwide are shown in Figures 3, 4, 5. Most Thai samples exhibited unique haplotypes found in single individuals, although some haplotypes were shared among individuals within the Thai Andaman Sea. Additionally, shared haplotypes were found between the Gulf of Thailand and the Thai Andaman Sea for two species: *S. attenuata* and *S. longirostris* . For *S. attenuata*, two samples from the Gulf of Thailand − Nakhon Si Thammarat (END194) and Narathiwat (END080) − shared a haplotype (Hap_1) with samples from Phuket and Phang Nga in the Thai Andaman Sea (Figure 3A). Globally, 116 haplotypes were identified from 179 *S. attenuate* sequences based on a 322 bp alignment (Figure 3B). Among these, five were restricted to Thai waters, while two others were shared with the East Tropical Pacific, India, and the western North Atlantic Ocean (Figure 3B). For *S. coeruleoalba*, five of 29 haplotypes (Hap_4, 8, 9, 12, 22) were shared among individuals within the Thai Andaman Sea (Figure 4A). Most of these haplotypes differed by one to five mutational steps, although haplotypes 3 and 11 showed more mutation steps at 13 and 17 sites, respectively (Figure 4A). A total of 107 haplotypes were identified from 167 global sequences across a 311 bp region (Figure 4B), with 22 found exclusively in the Thai Andaman Sea. For *S. longirostris*, one sample from Surat Thani (END989) in the Gulf of Thailand shared the same haplotype (Hap_19) with a sample from Krabi (END2401) in the Thai Andaman Sea (Figure 5A). Globally, 129 haplotypes were identified from 229 sequences (Figure 5B). Of these, 22 haplotypes were unique to Thai samples, while another 11 haplotypes were shared with individuals from the Pacific, Indian, and Atlantic Oceans.

**FIGURE 6:**
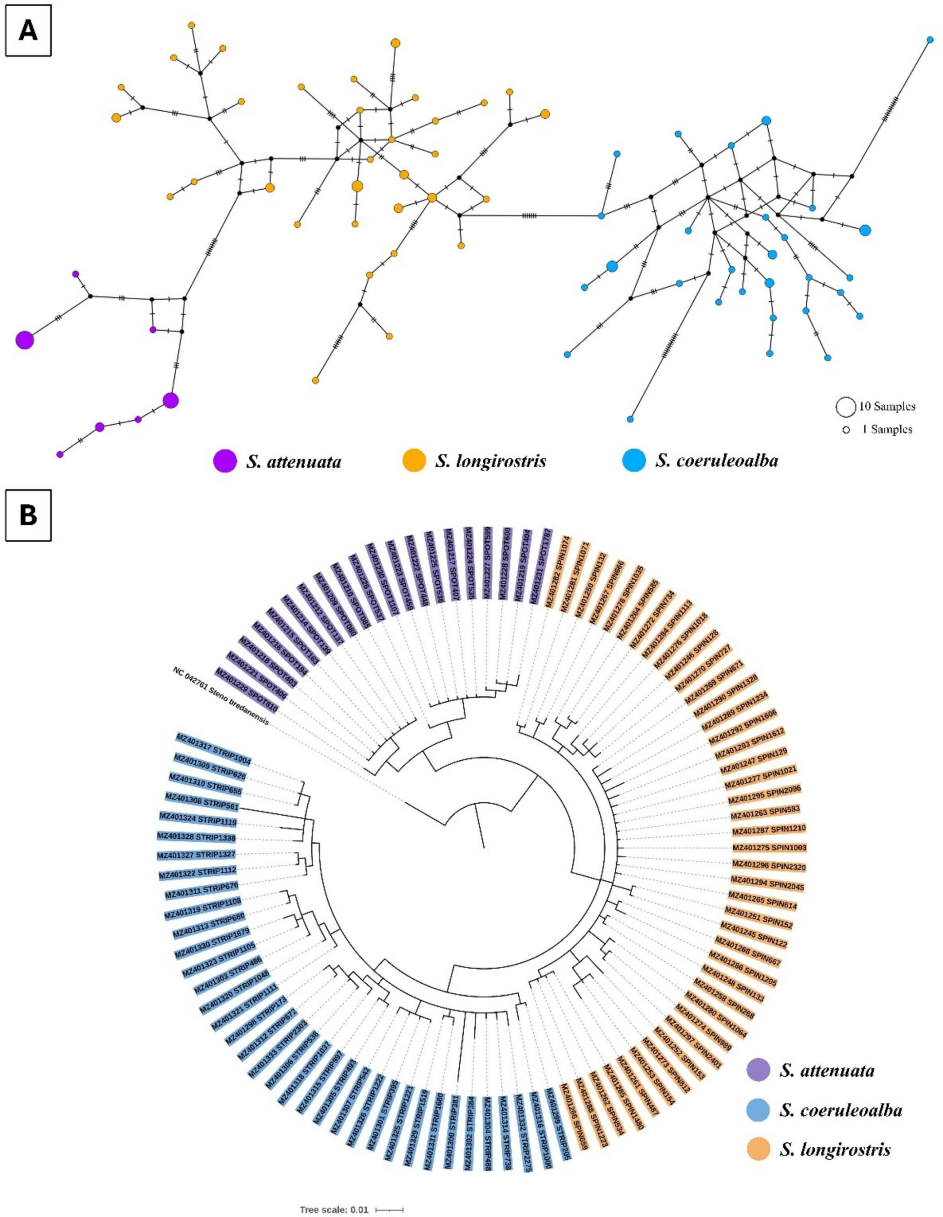
The median joining networks (MJNs) and inter‐specific phylogenetic tree of *Stenella* spp. from Thailand generated by 425 bp of 98 mtDNA control region sequences of *S. attenuata* (*n* = 20/23), *S. coeruleoalba* (*n* = 36/37), and *S. longirostris* (*n* = 42/53). (A) The MJNs showed three haplogroups of *Stenella* spp. Haplogroups are colored by species. Small black circles represent inferred haplotypes (not sampled). The size of each circle is proportional to its haplotype frequency, while nucleotide substitutions are shown at the branches as small transverse bars. (B) Interspecific phylogenetic tree indicated three clades of *Stenella* spp. from Thailand. The samples were labeled by accession number. The color indicated clades of each species.

The haplotype relationships among the three *Stenella* species are illustrated in the MJNs (Figure 6A). Each species formed a distinct cluster, with no haplotype sharing observed among them. Genetic differentiation was evident, with seven mutation steps separating *S. coeruleoalba* and *S. longirostris* , and nine steps between *S. attenuata* and *S. coeruleoalba* . These differences resulted in three distinct haplogroups, each corresponding to a single species (Figure 6A). The global distribution of haplotypes across the three species is shown in Figure S1. A total of 575 sequences from all mtDNA datasets were combined, resulting in 327 unique haplotypes based on a 307 bp alignment. All samples from this study clustered within their respective species‐specific haplogroups, with no evidence of haplotype sharing across species. Bayesian phylogenetic reconstruction further supported these patterns, revealing a polyphyletic grouping of *Stenella* spp. (Figure 6B). Three distinct monophyletic clades representing each species from Thai waters were observed. Within these clades, *S. attenuata* was further divided into two sub‐clades, while *S. coeruleoalba* and *S. longirostris* formed closely related sister taxa (Figure 6B).

**FIGURE 7:**
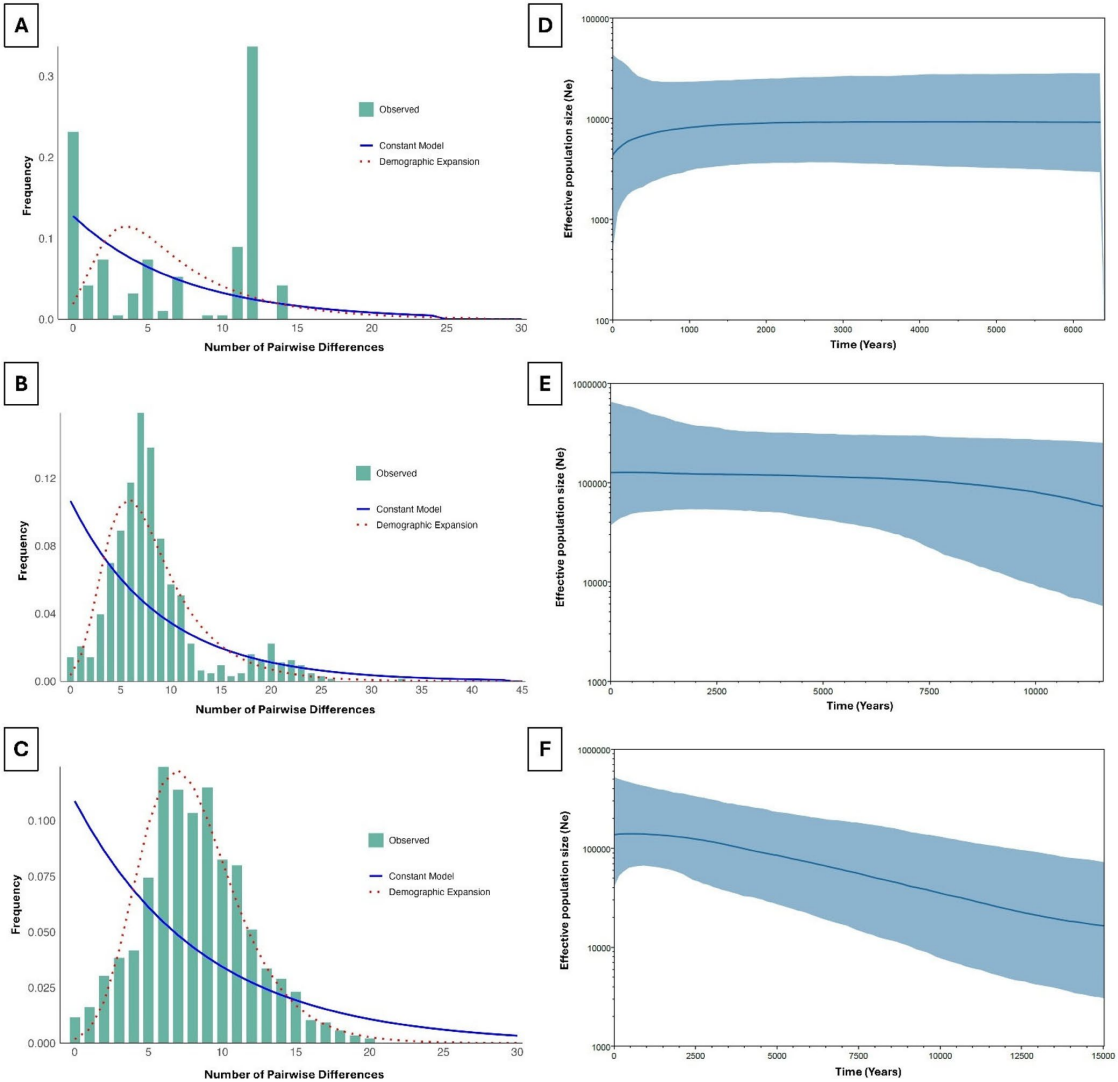
Mismatch distribution analysis (A‐C) and Bayesian Skyline Plots (BSPs) (D‐F) of three *Stenella* spp. populations in Thai waters. Mismatch distribution showing (A) bimodal distribution in *S. attenuata* , (B) unimodal in *S. coeruleoalba*, and (C) unimodal in *S. longirostris* . Bars indicate the frequency of pairwise nucleotide differences between individuals. Curves correspond to the expected distribution fitted to the data under a model of constant population size (solid blue line) or demographic expansion (dotted red line). BSPs illustrate historical demographic trends of (D) *S. attenuata*, (E) *S. coeruleoalba*, and (F) *S. longirostris* . Estimates of effective population size (Ne) are shown as a solid line, with shaded areas representing the 95% highest posterior density (HPD) interval.

Neutrality tests, mismatch distribution analyses, and BSPs revealed distinct demographic histories among the three *Stenella* species (Figure 7). For *S. attenuata* , both Tajima's D (1.93, *p* = 0.984) and Fu's Fs (2.89, *p* = 0.900) were positive and non‐significant, indicating no evidence of recent population expansion. This finding is consistent with the observed bimodal mismatch distribution (Figure 7A), suggesting a stable or possibly structured population. Although the raggedness index was 0 (*p* = 0.000), which typically suggests population expansion, this result should be interpreted with caution due to the small sample size. The BSP analysis also indicated prolonged demographic stability, followed by a slight population decline beginning approximately 1000 years ago (Figure 7D). In *S. coeruleoalba* , Tajima's D was slightly negative but not significant (−1.29, *p* = 0.078), while Fu's Fs was significantly negative (−16.14, *p* = 0.000), indicating a potential signal of population expansion. The unimodal mismatch distribution and a raggedness index of 0 (*p* = 0.000) also support this interpretation (Figure 7B). The BSP showed signs of slow expansion before 10,000 years ago, stabilizing around 2500–5000 years ago (Figure 7E). In contrast, *S. longirostris* showed strong evidence of recent demographic expansion, with a significantly negative Fu's Fs value (−20.11, *p* = 0.000), a slightly negative but non‐significant Tajima's D (−1.14, *p* = 0.133), a unimodal mismatch distribution (Figure 7C), and a very low raggedness index (0.0067, *p* = 0.004). The BSP also showed clear demographic expansion starting around 15,000 years ago (Figure 7F).

## 4 Discussion

Overall, the high genetic diversity observed in both nDNA microsatellite and mtDNA control region analyses aligns with existing population estimates, supporting the conclusion that *Stenella* spp. are among the most abundant cetacean species in the Thai Andaman Sea (Central Database System and Data Standard for Marine and Coastal Resources 2013a). The microsatellite data further indicate that *Stenella* species exhibit greater genetic diversity than other Thai cetacean populations reported in previous studies. For example, Dai et al. (2022) assessed genetic diversity in Indo‐Pacific humpback dolphins ( *Sousa chinensis* ) using 18 microsatellite loci and reported *Ho* values of 0.497 and 0.442, and *Na* of 3.11 and 4.15 in the Thai Andaman Sea and Western Gulf of Thailand, respectively. Similarly, low genetic diversity has been documented in Irrawaddy dolphins ( *Orcaella brevirostris* ) from Songkhla Lake and the Lower Gulf of Thailand. In the Songkhla lake population, Budi et al. (2024) reported *Na* and *AR* values of just 1.18 and 1.12, and an *Ho* as low as 0.039. These genetic patterns align with the critically low estimated population size in the lake—fewer than 20 individuals remain (Central Database System and Data Standard for Marine and Coastal Resources 2024). However, for Indo‐Pacific bottlenose dolphin ( *Tursiops aduncus* ), using 11 loci, the mean *Na* and *Ho* values were at 7.82 and 0.64 (Prasitwiset et al. 2022), similar to *Stenella* spp. in our study. While the high genetic diversity observed in *Stenella* species may reflect relatively robust and stable populations, caution is advised when making direct comparisons across studies, as differences in the number, type, and species‐specificity of loci can influence diversity estimates. Moreover, across all three *Stenella* spp. in our study, *Fis* values were close to zero. Although positive values were observed in *S. attenuata* and *S. longirostris* , the lack of significant deviations from HWE across all loci suggests that these *Fis* do not reflect true population inbreeding, but rather normal variability among loci, supporting the conclusion that the studied populations maintain genetic stability and diversity, with no clear signs of genetic stress.

Beyond Thai waters, the genetic status and population structure of *Stenella* spp. have also been studied in other regions, including Hawaiian waters, East Tropical Pacific, the Atlantic Ocean, and the Mediterranean Sea (Escorza‐Trevino et al. 2005; Courbis et al. 2014; Gaspari et al. 2019; Andrews et al. 2010; Faria et al. 2022). In the eastern tropical Pacific, seven microsatellite loci were used to study the genetic diversity of *S. attenuata* inhabiting coastal and offshore areas from North Mexico to Ecuador (Escorza‐Trevino et al. 2005). The mean allelic richness per locus ranged from 3.67 to 4.28, which is lower than the values from our study. Additionally, two genetically distinct populations of *S. attenuata* in East Tropical Pacific were identified as coastal and offshore populations, suggesting they should be treated as separate management units (Escorza‐Trevino et al. 2005). In contrast, a recent study of *S. attenuata* in Hawaiian waters using 11 microsatellite loci revealed higher levels of heterozygosity (Courbis et al. 2014). The *Na* ranged from 6 to 20, and *He* ranged from 0.794 to 0.841 (Courbis et al. 2014). For *S. coeruleoalba* , a population study by Gaspari et al. (2019) using 15 microsatellite loci suggested that separate populations exist between the Atlantic Ocean and Mediterranean Sea. Similarly, genetic analysis of *S. longirostris* in the Fernando de Noronha Archipelago off the northeast coast of Brazil used 10 microsatellite loci and revealed high *Ho* (0.767) but no evidence of genetic substructure (Faria et al. 2020, 2022). However, mtDNA analysis of the same population revealed lower haplotype diversity (*H* = 0.374) and at least two distinct maternal lineages that were highly differentiated from other global populations—findings that may reflect strong site fidelity, particularly among females (Faria et al. 2020, 2022). While previous studies have utilized varying numbers of microsatellite loci, ranging from seven to 15, and reported differing levels of population structure among *Stenella* species, our analysis employing 8, 9, and 14 loci for *S. attenuata, S. coeruleoalba*, and *S. longirostris* , respectively, revealed no significant genetic structuring within Thai waters. Given that the loci used in this study exhibited sufficient polymorphism and discriminatory power, as indicated by robust PID, PIDsib, and PIC values, the lack of detectable population structure is unlikely attributable to marker limitations. It is possible, however, that the relatively small sample sizes may have reduced the ability to detect subtle population differentiation.

In our study, *S. attenuata* exhibited the highest *He* based on microsatellite data, yet showed the lowest *Hd* and *π* from mtDNA analyses. The mtDNA diversity of *S. attenuata* was also notably lower than that reported for *Tursiops aduncus* in the Thai Andaman Sea, where Prasitwiset et al. (2022) identified 17 haplotypes (*H* = 0.926 ± 0.027, *π* = 0.045 ± 0.002) from 30 individuals using a 232 bp fragment of the mtDNA control region. This lower mtDNA diversity in *S. attenuata* may reflect a high degree of site fidelity, particularly among females, which has also been shown for other populations around the Hawaiian Islands (Courbis et al. 2014). Such long‐term residency in a localized area may have restricted maternal gene flow between Thai and non‐Thai populations, leading to reduced mtDNA variation within those regions. Habitat fidelity in *Stenella* spp. has similarly been documented in other locations, including *S. attenuata* in Guadeloupe and Martinique (Courtin et al. 2023) and *S. longirostris* in Moon reef, Fiji Islands (Cribb et al. 2012). However, the extent of site fidelity appears to vary among *Stenella* populations and locations (Andrews et al. 2010).

In Hawaiian waters, only 10 haplotypes of *S. attenuata* were identified among 100 individuals, with 77 sharing a single dominant haplotype (Courbis et al. 2014). Despite this low haplotype diversity, significant genetic differences were found among regional populations from Hawaii, Maui/Lanai, and Oahu, possibly due to site fidelity and behavioral isolation (Courbis et al. 2014). In contrast, *S. attenuata* populations in the eastern tropical Pacific exhibited higher mtDNA diversity, with 112 haplotypes identified among 225 individuals (Escorza‐Trevino et al. 2005). Although some haplotypes were shared between coastal and offshore populations, significant genetic differences were still evident (Escorza‐Trevino et al. 2005). In Thai waters, *Stenella* populations are primarily known from offshore sightings and stranding events, with no evidence to date of island‐associated or resident coastal populations (Central Database System and Data Standard for Marine and Coastal Resources 2013b, 2013c, 2013d; Adulyanukosol et al. 2012; Chantrapornsyl et al. 1996). However, unique haplotypes restricted to Thai waters were detected in all species, which may imply the distinct population that is not related to others. This finding warrants careful monitoring, and further study of population site fidelity is needed. The lower mtDNA diversity and absence of detected population structure in *S. attenuata* should be interpreted with caution, as the limited sample size may have influenced these results. Additionally, caution is warranted when interpreting population structure and site fidelity from stranded samples, as they may not capture the full diversity or residency behaviors present in the live populations. Inclusion of more individuals in future studies and using biopsy techniques will be essential to monitor population diversity and explore potential population structure.

Strong evidence of female philopatry was observed across the three *Stenella* species in Thai waters, as evidenced by the complete absence of shared mitochondrial haplotypes among them. This pattern indicates highly restricted maternal gene flow, despite the overlapping distribution of these species. In contrast, nuclear microsatellite data revealed traces of admixture in several individuals, specifically *S. coeruleoalba* (END205, END395, END2303, and END1108) and *S. longirostris* (END1330 and END812), whose genetic profiles showed partial affinities with those of other *Stenella* species. These individuals maintained their species‐specific mitochondrial haplotypes and morphological traits, yet exhibited nuclear evidence of interspecific genetic mixing. Such a discrepancy between maternally inherited mtDNA and biparentally inherited nuclear DNA strongly supports the hypothesis that males are the primary vectors of gene flow among these species. Consequently, male‐driven gene flow may explain the absence of clear population structure within species based on nuclear DNA markers. This pattern aligns with findings in other cetaceans, where male‐biased dispersal has been documented, such as in the Indo‐Pacific bottlenose dolphin ( *Tursiops aduncus* ) and the sperm whale ( *Physeter macrocephalus* ), while females show greater philopatry (Engelhaupt et al. 2009; Möller and Beheregaray 2004; Dulau et al. 2017). Similar cases of interspecific hybridization within *Stenella* ( *S. clymene* and *S. coeruleoalba* ) have been reported in Brazilian waters, where individuals morphologically assigned to one species carried mtDNA from another, suggesting maternal introgression (Faria et al. 2022). Additionally, *S. longirostris* × *S. attenuata* hybrids have been observed in the Fernando de Noronha archipelago, with individuals displaying morphological features of both species (Silva et al. 2005). However, our study presents a contrasting scenario in which nuclear, rather than mitochondrial, evidence suggests gene flow, highlighting male‐mediated introgression as a dominant force shaping the nuclear genetic structure of *Stenella* populations in Thai waters. Currently, no mixed‐species groups involving multiple *Stenella* species have been documented in Thai waters. However, long‐term monitoring programs may provide further insight into the frequency and behavioral dynamics of potential interspecific associations, which could help clarify the mechanisms underlying the observed genetic admixture.

The demographic history of *Stenella* spp., particularly the three species examined in this study, remains poorly understood. Thus, the natural and historical events and recent anthropogenic impacts cannot be excluded as potential drivers of the observed changes in population structure and genetic diversity. Faria et al. (2020) suggested that the loss of mitochondrial haplotypes in Noronha spinner dolphins could be a result of genetic drift, restricted female‐mediated gene flow, and a small founding population, which is consistent with a historical bottleneck. These differences may reflect species‐specific responses to environmental change following the Last Glacial Maximum, as well as varying sensitivity to recent anthropogenic impacts (Steeman et al. 2009; Daochai et al. 2024). The clear signal of demographic expansion in *S. longirostris*, beginning approximately 15,000 years ago, coincides with postglacial sea‐level rise around 12,500 to 16,000 years ago (Lambeck and Chappell 2001), which dramatically reshaped marine habitats in Southeast Asia, including the Thai Andaman Sea and the Gulf of Thailand (Solihuddin 2014). This expansion suggests that *S. longirostris* may have rapidly colonized and benefited from these newly available ecological niches. A similar expansion pattern, followed by stabilization, was observed in *S. coeruleoalba* . In contrast, *S. attenuata* displayed long‐term demographic stability followed by a recent decline (1000 years ago), possibly linked to more recent climate change or anthropogenic pressures, a trend not observed in the other two species. The linear pattern of MJNs (Figure 3A) and limited number of polymorphic sites further support a history of population contraction or slow mutation accumulation in Thai *S. attenuata* . These divergent demographic patterns suggest that *Stenella* species in Thai waters may vary in their sensitivity and resilience to environmental change. Differences in ecological preferences, behavioral plasticity, and interactions with human activities (e.g., prey availability, fishing pressure, and climate variability) could have contributed to these species‐specific trajectories. Further research is warranted to better understand how such factors shape population dynamics and to inform conservation strategies tailored to the unique ecological responses of each species in this region.

## 5 Conclusion

This study advances our understanding of the genetic diversity, population structure, and interspecific maternal lineage relationships among three dolphins in the genus *Stenella* ( *S. longirostris* , *S. coeruleoalba* , *S. attenuata* ) through analyses of multiple loci of microsatellite nDNA and mtDNA markers. These species exhibited high levels of genetic diversity for both multi‐loci of microsatellite nDNA and mtDNA control regions, with *S. attenuata* showing the highest observed heterozygosity of nDNA microsatellite and *S. longirostris* showing the highest mtDNA diversity. Although no significant population structure was detected within individual species, analysis of the combined dataset revealed clear genetic differentiation and evidence of admixture among species. While there were no shared mtDNA haplotypes among the species, several individuals showed signs of interspecific hybridization based on microsatellite data. Unique mtDNA haplotypes were also identified in Thai waters for all three species, underscoring the region's genetic distinctiveness. Demographic analyses revealed species‐specific histories likely shaped by past environmental shifts and recent anthropogenic pressures. *S. longirostris* and *S. coeruleoalba* showed signatures of historical population expansion, likely linked to postglacial sea‐level rise, whereas *S. attenuata* exhibited long‐term demographic stability followed by a more recent decline. These contrasting patterns suggest differing levels of sensitivity and resilience to environmental change. Given their overlapping distributions and genetic distinctiveness, ongoing monitoring of these species is warranted. Future research should focus on investigating fine‐scale population structure to inform conservation strategies that preserve genetic diversity among these sympatric dolphin populations in Thai waters. This will require studies with larger sample sizes to enhance the robustness of analyses and interpretations. Additionally, genome‐wide analyses are needed to detect intra‐species population structure in these highly mobile animals, particularly within localized areas.

## Author Contributions

**Promporn Piboon:** conceptualization (lead), data curation (equal), formal analysis (equal), funding acquisition (lead), investigation (lead), methodology (lead), project administration (equal), software (lead), validation (lead), writing – original draft (equal). **Janine L. Brown:** supervision (equal), validation (equal), writing – review and editing (equal). **Patcharaporn Kaewmong:** data curation (supporting), investigation (supporting), resources (lead), validation (supporting). **Kongkiat Kittiwattanawong:** data curation (supporting), investigation (supporting), resources (lead), validation (supporting). **Sarisa Klinhom:** investigation (equal). **Toshiaki Yamamoto:** supervision (supporting), validation (supporting). **Korakot Nganvongpanit:** conceptualization (equal), project administration (equal), supervision (equal), validation (equal), writing – original draft (equal), writing – review and editing (equal).

## Ethics Statement

This study was approved by the Animal Use Committee of the Faculty of Veterinary Medicine, Chiang Mai University, Thailand, in 2020 (S14/2563).

## Conflicts of Interest

The authors declare no conflicts of interest. The funders had no role in the design of the study, in the collection, analysis, or interpretation of data, in the writing of the manuscript, or in the decision to publish the results.

## Supporting information


**Figure S1:** The median joining networks (MJNs) of 327 haplotypes of *Stenella* spp. (*S. attenuata, S. coeruleoalba* and *S. longirostris* ) generated 575 sequences (307 bp). Each haplotype is colored by species. Purple is for *S. attenuata*, Orange is for *S. logirostris* and Blue is for *S. coeruleoalba*. The lighter shade is our samples. The small black circles represent inferred haplotypes (not sampled). The size of each circle is proportional to its haplotype frequency, while nucleotide substitutions are shown as 1‐step edges.

**Table S1:** All samples of *Stenella* species from Thai seas used in this study.

**Table S2:** The list of twenty microsatellite loci used in this study.

**Table S3:** All sequences and accession numbers used in this study.

**Table S4:** The detail of microsatellite loci *Stenella attenuata* used in this study.

**Table S5:** The detail of microsatellite loci *Stenella coeruleoalba* used in this study.

**Table S6:** The detail of microsatellite loci *Stenella longirostris* used in this study.

**Table S7:** The detail of most likely K value using a combined dataset (10 loci), *Stenella attenuata* (8 loci), *Stenella coeruleoalba* (9 loci) *and Stenella longirostris* (14 loci) from multiple criteria, including the Pritchard method (Pr[X|K]), the Evanno method (ΔK), and the Parsimony index (PI).

## Data Availability

The data generated during the current study is available in the Tables S1–, S7 and Figure S1.
