# Supplementary material for: Genetic Diversity and Inter‐Specific Phylogeny of Three Sympatric Cetacean Species (Stenella spp.) in Thai Territorial Waters Based on Mitochondrial and Nuclear DNA Markers
Source: Ecol Evol. 2025 Oct 12;15(10):e72322. doi: 10.1002/ece3.72322 (PMC12516012; doi:10.1002/ece3.72322)
Supplement: Supplementary file 1 — Figure S1: The median joining networks (MJNs) of 327 haplotypes of Stenella spp. ( S. attenuata, S. coeruleoalba and S. longirostris ) generated 575 sequences (307 bp). Each haplotype is colored by species. Purple is for S. attenuata, Orange is for S. logirostris and Blue is for S. coeruleoalba. The lighter shade is our samples. The small black circles represent inferred haplotypes (not sampled). The size of each circle is proportional to its haplotype frequency, while nucleotide substitutions are shown as 1‐step edges. [file ECE3-15-e72322-s008.docx]

**Genetic diversity and inter-specific phylogeny of three sympatric cetacean species (*Stenella* spp.) in Thai territorial waters based on mitochondrial and nuclear DNA markers**

Promporn Piboon^1^, Janine Brown^2^, Patcharaporn Kaewmong^3^, Kongkiat Kittiwattanawong^4^ Sarisa Klinhom^1^, Toshiaki Yamamoto^5^, and Korakot Nganvongpanit^1,^*

^1^ The School of Veterinary Medicine, Faculty of Veterinary Medicine, Chiang Mai University, Chiang Mai 50100, Thailand.

^2^ Smithsonian Conservation Biology Institute, Center for Species Survival, 1500 Remount Rd, Front Royal, VA, United States.

^3^ Phuket Marine Biological Center, Phuket 83000, Thailand.

^4^ Department of Marine and Coastal Resources, Ratthaprasasanabhakti Building (Building B) The Government Complex, Bangkok 10210, Thailand

^5^ Department of Veterinary Nursing and Technology, Nippon Veterinary and Life Science University, Musashino, Tokyo, Japan

* Correspondence: korakot.n@cmu.ac.th

E-mail:

PP = promporn.piboon@cmu.ac.th

JB= BrownJan@si.edu

PK = marineanimal.vet@gmail.com

KK = kkongkiat@gmail.com

SK= Yui.sarisarisa@gmail.com

TY= tyamamoto@nvlu.ac.jp

KN = korakot.n@cmu.ac.th


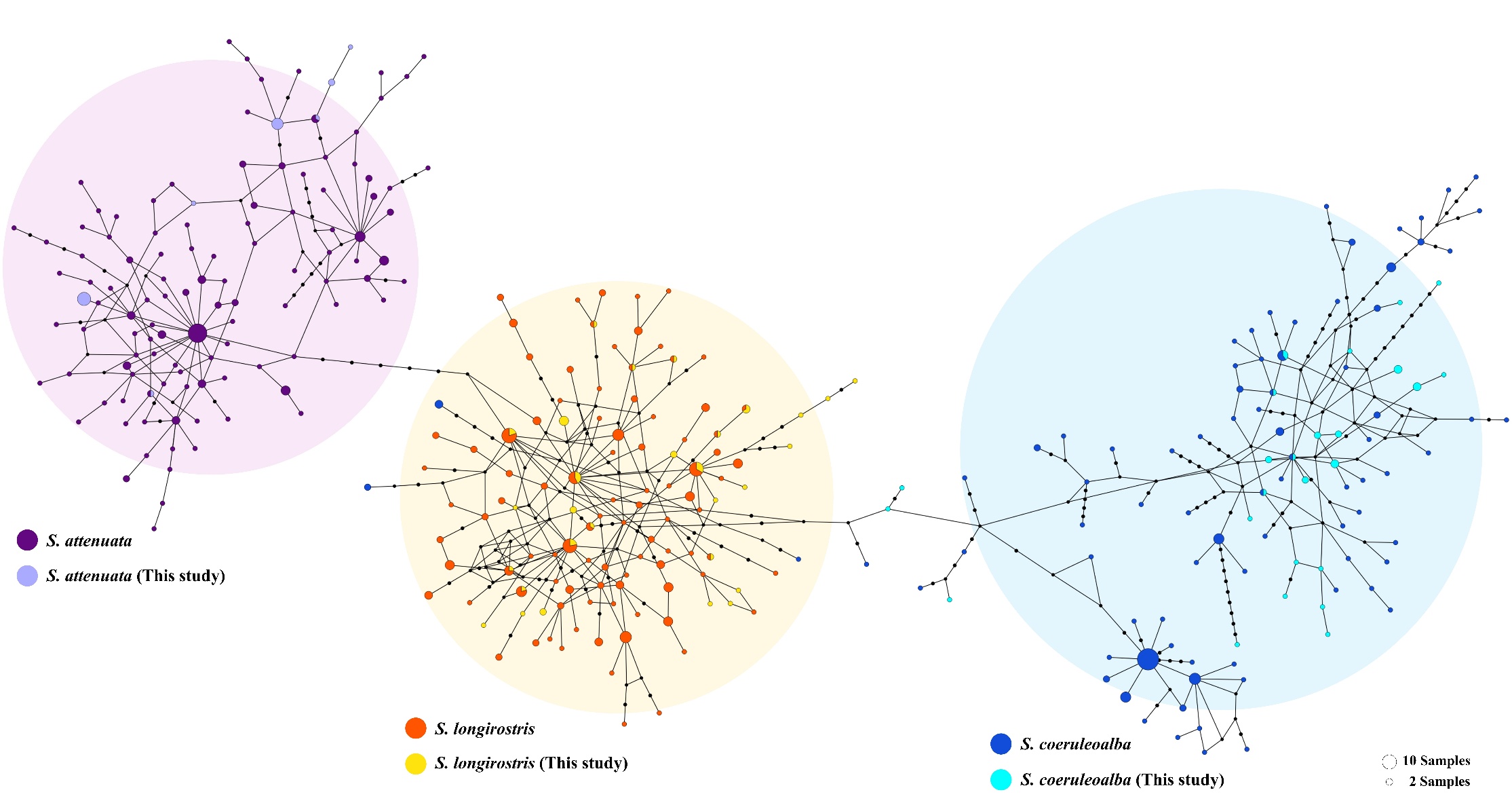


**Figure S1.** The median joining networks (MJNs) of 327 haplotypes of *Stenella* spp. (*S. attenuata, S. coeruleoalba* and *S. longirostris*) generated 575 sequences (307 bp). Each haplotype is colored by species*.* Purple is for *S. attenuata,* Orange is for *S. logirostris* and Blue is for *S. coeruleoalba.* The lighter shade is our samples. The small black circles represent inferred haplotypes (not sampled). The size of each circle is proportional to its haplotype frequency, while nucleotide substitutions are shown as 1-step edges.
