# Supplementary material for: Genetic Diversity and Inter‐Specific Phylogeny of Three Sympatric Cetacean Species (Stenella spp.) in Thai Territorial Waters Based on Mitochondrial and Nuclear DNA Markers
Source: Ecol Evol. 2025 Oct 12;15(10):e72322. doi: 10.1002/ece3.72322 (PMC12516012; doi:10.1002/ece3.72322)
Supplement: Supplementary file 2 — Table S1: All samples of Stenella species from Thai seas used in this study. [file ECE3-15-e72322-s001.docx]

**The genetic diversity and inter-specific phylogeny of three sympatric cetacean species (*Stenella* spp.) in Thai territorial waters based on mitochondrial and nuclear DNA markers**

Promporn Piboon^1^, Janine Brown^2^, Patcharaporn Kaewmong^3^, Kongkiat Kittiwattanawong^4^ Sarisa Klinhom^1^, Toshiaki Yamamoto^5^, and Korakot Nganvongpanit^1,^*

^1^ The School of Veterinary Medicine, Faculty of Veterinary Medicine, Chiang Mai University, Chiang Mai 50100, Thailand.

^2^ Smithsonian Conservation Biology Institute, Center for Species Survival, 1500 Remount Rd, Front Royal, VA, United States.

^3^ Phuket Marine Biological Center, Phuket 83000, Thailand.

^4^ Department of Marine and Coastal Resources, Ratthaprasasanabhakti Building (Building B) The Government Complex, Bangkok 10210, Thailand

^5^ Department of Veterinary Nursing and Technology, Nippon Veterinary and Life Science University, Musashino, Tokyo, Japan

* Correspondence: korakot.n@cmu.ac.th

E-mail:

PP = promporn.piboon@cmu.ac.th

JB= BrownJan@si.edu

PK = marineanimal.vet@gmail.com

KK = kkongkiat@gmail.com

SK= Yui.sarisarisa@gmail.com

TY= tyamamoto@nvlu.ac.jp

KN = korakot.n@cmu.ac.th

**Table S1.** All samples of *Stenella* species from Thai seas used in this study.

| **No** | **Sample code** | **Species** | **mtDNA Accession number** | **nDNA Microsatellite** | **Stranding location** |
| --- | --- | --- | --- | --- | --- |
| 1 | END810 | *Stenella attenuata* | MZ401229 | - | Krabi |
| 2 | END194 | *Stenella attenuata* | MZ401216 | YES | Nakornsithamarat |
| 3 | END080 | *Stenella attenuata* | MZ401209 | YES | Narathiwat |
| 4 | END407 | *Stenella attenuata* | MZ401217 | YES | Phangnga |
| 5 | END137 | *Stenella attenuata* | MZ401212 | YES | Phangnga |
| 6 | END403 | *Stenella attenuata* | MZ401218 | YES | Phangnga |
| 7 | END404 | *Stenella attenuata* | MZ401219 | YES | Phangnga |
| 8 | END406 | *Stenella attenuata* | MZ401221 | YES | Phangnga |
| 9 | END459 | *Stenella attenuata* | MZ401223 | YES | Phuket |
| 10 | END1107 | *Stenella attenuata* | MZ401230 | YES | Phuket |
| 11 | END119 | *Stenella attenuata* | - | YES | Phuket |
| 12 | END537 | *Stenella attenuata* | MZ401226 | YES | Phuket |
| 13 | END599 | *Stenella attenuata* | MZ401227 | YES | Phuket |
| 14 | END600 | *Stenella attenuata* | MZ401228 | YES | Phuket |
| 15 | END1787 | *Stenella attenuata* | MZ401231 | YES | Phuket |
| 16 | END535 | *Stenella attenuata* | MZ401224 | YES | Trang |
| 17 | END448 | *Stenella attenuata* | MZ401222 | - | Phuket |
| 18 | END536 | *Stenella attenuata* | MZ401225 | - | Phuket |
| 19 | END085 | *Stenella attenuata* | MZ401210 | - | Phangnga |
| 20 | END138 | *Stenella attenuata* | - | - | Phangnga |
| 21 | END139 | *Stenella attenuata* | MZ401214 | - | Phangnga |
| 22 | END163 | *Stenella attenuata* | MZ401215 | - | Phuket |
| 23 | END405 | *Stenella attenuata* | - | - | Phangnga |
| 24 | END1680 | *Stenella coeruleoalba* | MZ401331 | YES | Krabi |
| 25 | END381 | *Stenella coeruleoalba* | MZ401300 | YES | Phangnga |
| 26 | END384 | *Stenella coeruleoalba* | MZ401302 | YES | Phangnga |
| 27 | END486 | *Stenella coeruleoalba* | MZ401303 | YES | Phangnga |
| 28 | END538 | *Stenella coeruleoalba* | MZ401306 | YES | Phangnga |
| 29 | END1004 | *Stenella coeruleoalba* | MZ401317 | YES | Phangnga |
| 30 | END1037 | *Stenella coeruleoalba* | MZ401318 | YES | Phangnga |
| 31 | END1111 | *Stenella coeruleoalba* | MZ401321 | YES | Phangnga |
| 32 | END2275 | *Stenella coeruleoalba* | MZ401332 | YES | Phangnga |
| 33 | END488 | *Stenella coeruleoalba* | MZ401304 | YES | Phuket |
| 34 | END2273 | *Stenella coeruleoalba* | - | - | Phuket |
| 35 | END655 | *Stenella coeruleoalba* | MZ401310 | - | Phuket |
| 36 | END738 | *Stenella coeruleoalba* | MZ401314 | YES | Phuket |
| 37 | END173 | *Stenella coeruleoalba* | MZ401298 | YES | Phuket |
| 38 | END205 | *Stenella coeruleoalba* | MZ401299 | YES | Phuket |
| 39 | END395 | *Stenella coeruleoalba* | MZ401301 | YES | Phuket |
| 40 | END542 | *Stenella coeruleoalba* | MZ401307 | YES | Phuket |
| 41 | END670 | *Stenella coeruleoalba* | MZ401311 | YES | Phuket |
| 42 | END672 | *Stenella coeruleoalba* | MZ401312 | YES | Phuket |
| 43 | END997 | *Stenella coeruleoalba* | MZ401315 | YES | Phuket |
| 44 | END1119 | *Stenella coeruleoalba* | MZ401324 | YES | Phuket |
| 45 | END1327 | *Stenella coeruleoalba* | MZ401327 | YES | Phuket |
| 46 | END1519 | *Stenella coeruleoalba* | MZ401329 | YES | Phuket |
| 47 | END1679 | *Stenella coeruleoalba* | MZ401330 | YES | Phuket |
| 48 | END626 | *Stenella coeruleoalba* | MZ401309 | YES | Ranong |
| 49 | END493 | *Stenella coeruleoalba* | MZ401305 | YES | Satun |
| 50 | END1000 | *Stenella coeruleoalba* | MZ401316 | YES | Satun |
| 51 | END2303 | *Stenella coeruleoalba* | MZ401333 | YES | Satun |
| 52 | END1108 | *Stenella coeruleoalba* | MZ401319 | YES | Satun |
| 53 | END561 | *Stenella coeruleoalba* | MZ401308 | - | Trang |
| 54 | END680 | *Stenella coeruleoalba* | MZ401313 | YES | Trang |
| 55 | END1048 | *Stenella coeruleoalba* | MZ401320 | YES | Trang |
| 56 | END1105 | *Stenella coeruleoalba* | MZ401323 | YES | Trang |
| 57 | END1112 | *Stenella coeruleoalba* | MZ401322 | YES | Trang |
| 58 | END1221 | *Stenella coeruleoalba* | MZ401325 | YES | Trang |
| 59 | END1222 | *Stenella coeruleoalba* | MZ401326 | YES | Trang |
| 60 | END1338 | *Stenella coeruleoalba* | MZ401328 | YES | Trang |
| 61 | END122 | *Stenella longirostris* | MZ401245 | YES | Ranong |
| 62 | END128 | *Stenella longirostris* | MZ401246 | - | Trang |
| 63 | END129 | *Stenella longirostris* | MZ401247 | YES | Trang |
| 64 | END133 | *Stenella longirostris* | MZ401248 | - | Trang |
| 65 | END132 | *Stenella longirostris* | MZ401250 | YES | Trang |
| 66 | END152 | *Stenella longirostris* | MZ401251 | - | Phuket |
| 67 | END153 | *Stenella longirostris* | MZ401252 | - | Phuket |
| 68 | END154 | *Stenella longirostris* | MZ401253 | YES | Phuket |
| 69 | END268 | *Stenella longirostris* | MZ401258 | YES | Phuket |
| 70 | END487 | *Stenella longirostris* | MZ401261 | YES | Phuket |
| 71 | END534 | *Stenella longirostris* | MZ401262 | YES | Phuket |
| 72 | END583 | *Stenella longirostris* | MZ401263 | YES | Phuket |
| 73 | END585 | *Stenella longirostris* | MZ401264 | YES | Phuket |
| 74 | END614 | *Stenella longirostris* | MZ401265 | YES | Phuket |
| 75 | END659 | *Stenella longirostris* | MZ401266 | YES | Phuket |
| 76 | END666 | *Stenella longirostris* | MZ401267 | YES | Phuket |
| 77 | END667 | *Stenella longirostris* | MZ401268 | YES | Krabi |
| 78 | END671 | *Stenella longirostris* | MZ401269 | YES | Phuket |
| 79 | END727 | *Stenella longirostris* | MZ401270 | YES | Nakornsithamarat |
| 80 | END734 | *Stenella longirostris* | MZ401272 | YES | Phuket |
| 81 | END812 | *Stenella longirostris* | MZ401273 | YES | krabi |
| 82 | END989 | *Stenella longirostris* | MZ401274 | YES | Suratthani |
| 83 | END1003 | *Stenella longirostris* | MZ401275 | YES | Phuket |
| 84 | END1018 | *Stenella longirostris* | MZ401276 | YES | Phangnga |
| 85 | END1021 | *Stenella longirostris* | MZ401277 | YES | Phuket |
| 86 | END1035 | *Stenella longirostris* | MZ401278 | YES | Krabi |
| 87 | END1064 | *Stenella longirostris* | MZ401280 | YES | Phuket |
| 88 | END1071 | *Stenella longirostris* | MZ401281 | YES | Phuket |
| 89 | END1074 | *Stenella longirostris* | MZ401282 | YES | Phangnga |
| 90 | END1113 | *Stenella longirostris* | MZ401284 | YES | Krabi |
| 91 | END1480 | *Stenella longirostris* | MZ401285 | YES | Phangnga |
| 92 | END1205 | *Stenella longirostris* | MZ401286 | YES | Trang |
| 93 | END1210 | *Stenella longirostris* | MZ401287 | YES | Phuket |
| 94 | END1223 | *Stenella longirostris* | MZ401288 | YES | Phuket |
| 95 | END1234 | *Stenella longirostris* | MZ401289 | - | Phangnga |
| 96 | END1328 | *Stenella longirostris* | MZ401290 | YES | Phuket |
| 97 | END1606 | *Stenella longirostris* | MZ401292 | YES | Chonburi |
| 98 | END1612 | *Stenella longirostris* | MZ401293 | YES | Phangnga |
| 99 | END2045 | *Stenella longirostris* | MZ401294 | YES | Phuket |
| 100 | END2096 | *Stenella longirostris* | MZ401295 | YES | Ranong |
| 101 | END2320 | *Stenella longirostris* | MZ401296 | YES | Trang |
| 102 | END2401 | *Stenella longirostris* | MZ401297 | YES | Krabi |
| 103 | END1330 | *Stenella longirostris* | - | YES | Phuket |
| 104 | END1097 | *Stenella longirostris* | - | YES | Phuket |
| 105 | END147 | *Stenella longirostris* | - | YES | Phuket |
| 106 | END172 | *Stenella longirostris* | - | - | Satun |
| 107 | END179 | *Stenella longirostris* | - | - | Phuket |
| 108 | END192 | *Stenella longirostris* | - | - | Phuket |
| 109 | END211 | *Stenella longirostris* | - | - | Phuket |
| 110 | END273 | *Stenella longirostris* | - | - | Phuket |
| 111 | END387 | *Stenella longirostris* | - | - | Phangnga |
| 112 | END729 | *Stenella longirostris* | - | - | Ranong |
| 113 | END1036 | *Stenella longirostris* | - | - | Phangnga |
