# Supplementary material for: Genetic Diversity and Inter‐Specific Phylogeny of Three Sympatric Cetacean Species (Stenella spp.) in Thai Territorial Waters Based on Mitochondrial and Nuclear DNA Markers
Source: Ecol Evol. 2025 Oct 12;15(10):e72322. doi: 10.1002/ece3.72322 (PMC12516012; doi:10.1002/ece3.72322)
Supplement: Supplementary file 3 — Table S2: The list of twenty microsatellite loci used in this study. [file ECE3-15-e72322-s002.docx]

**The genetic diversity and inter-specific phylogeny of three sympatric cetacean species (*Stenella* spp.) in Thai territorial waters based on mitochondrial and nuclear DNA markers**

Promporn Piboon^1^, Janine Brown^2^, Patcharaporn Kaewmong^3^, Kongkiat Kittiwattanawong^4^ Sarisa Klinhom^1^, Toshiaki Yamamoto^5^, and Korakot Nganvongpanit^1,^*

^1^ The School of Veterinary Medicine, Faculty of Veterinary Medicine, Chiang Mai University, Chiang Mai 50100, Thailand.

^2^ Smithsonian Conservation Biology Institute, Center for Species Survival, 1500 Remount Rd, Front Royal, VA, United States.

^3^ Phuket Marine Biological Center, Phuket 83000, Thailand.

^4^ Department of Marine and Coastal Resources, Ratthaprasasanabhakti Building (Building B) The Government Complex, Bangkok 10210, Thailand

^5^ Department of Veterinary Nursing and Technology, Nippon Veterinary and Life Science University, Musashino, Tokyo, Japan

* Correspondence: korakot.n@cmu.ac.th

E-mail:

PP = promporn.piboon@cmu.ac.th

JB= BrownJan@si.edu

PK = marineanimal.vet@gmail.com

KK = kkongkiat@gmail.com

SK= Yui.sarisarisa@gmail.com

TY= tyamamoto@nvlu.ac.jp

KN = korakot.n@cmu.ac.th

**Table S2.** The list of twenty microsatellite loci used in this study.

| **No.** | **Loci-Fluorescent dye** | **Primers sequences (5'-3')** | **Ta (^o^C)/ Status** | | | **Combined data set** | **References** |
| --- | --- | --- | --- | --- | --- | --- | --- |
|  |  |  | ***S. attenuata*** | ***S. coeruleoalba*** | ***S. longirostris*** |  |  |
| 1 | EV14-ROX | F-TAAACATCAAAGCAGACCCC | 64 ^o^C/ null allele | Incomplete amplification | - | - | Escorza-Trevino, et al. 2005[1] |
|  |  | R-CCAGAGCCAAGGTCAAGAG |  |  |  |  |  |
| 2 | EV37-HEX | F-AGCTTGATTTGGAAGTCATGA | 50 ^o^C/ null allele | - | 50 ^o^C | - |  |
|  |  | R-TAGTAGAGCCGTGATAAAGTGC |  |  |  |  |  |
| 3 | EV94-ROX | F-ATCGTATTGGTCCTTTTCTGC | - | 55 ^o^C/ null allele | 55 ^o^C | - |  |
|  |  | R-AATAGATAGTGATGATGATTCACACC |  |  |  |  |  |
| 4 | EV104-HEX | F-TGGAGATGACAGGATTTGGG | 43 ^o^C | 43 ^o^C | 43 ^o^C | YES |  |
|  |  | R-GGAATTTTTATTGTAATGGGTCC |  |  |  |  |  |
| 5 | Slo1-FAM | F-CAAACCAAAAGCAAACACACAC | Incomplete amplification | 63 ^o^C/ null allele | Incomplete amplification | - | Farro, et al. 2008[2] |
|  |  | R-CATCTCTATCAGCCATGTCCAA |  |  |  |  |  |
| 6 | Slo4-FAM | F-TAAGGTGAGAGGAGGTGGTGAT | 55 ^o^C | 55 ^o^C | 55 ^o^C | YES |  |
|  |  | R-CACGGTTGAGAGAATACAGGAA |  |  |  |  |  |
| 7 | Slo9-HEX | F-CCTTCTCCTCCTTCTGTCCTTT | 59 ^o^C / monomorphic | 59 ^o^C | 59 ^o^C | YES |  |
|  |  | R-CTTCCCTCACTTTCCCTACCC |  |  |  |  |  |
| 8 | Slo15-HEX | F-CGTCAAACTCCATCAAGACATC | - | 54 ^o^C | 54 ^o^C | - |  |
|  |  | R-ATCTCCACCACAAGACACCAC |  |  |  |  |  |
| 9 | Sco 11-HEX | F-ACCGCCTCTGTCTGTTTCTC | - | 55 ^o^C | 55 ^o^C | - | Mirimin, et al. 2006[3] |
|  |  | R-AAGTCACTCGGAGGAGTCCA |  |  |  |  |  |
| 10 | Sco 28-ROX | F-AAACCATTCCATTTTGAGGTAA | 55 ^o^C | 55 ^o^C | 55 ^o^C | YES |  |
|  |  | R-CCCTAGTATAAGAACATGGGAAGA |  |  |  |  |  |
| 11 | Sco 55-ROX | F-TGCAATTGGAGGTATCAGTGT | - | 55 ^o^C/ null allele | 55 ^o^C | - |  |
|  |  | R-GGTGTTTGGTGGTTGAGCAT |  |  |  |  |  |
| 12 | Sco 65-ROX | F-TACCTTGCACATTTTGGACAT | 55 ^o^C/ monomorphic | 55 ^o^C | 55 ^o^C/ monomorphic | YES |  |
|  |  | R-ATTAGTCAGGGTTCGCCATAG |  |  |  |  |  |
| 13 | Sco 66-TAMRA | F-AAAATGGCCAGTTGGGAAA | 55 ^o^C | 55 ^o^C | Incomplete amplification | YES |  |
|  |  | R-AAAATTTTCCCATGCAATAGA |  |  |  |  |  |
| 14 | Sd8-FAM | F-TGGCCGTTATAAATAGAGC | - | - | 52 ^o^C | - | Faria, et al. 2022[4] |
|  |  | R-GACAACAGTTTGGCAGTG |  |  |  |  |  |
| 15 | Sl1-25-TAMRA | F-TTGATTTTCTGACTTCTTGGG | 54 ^o^C | 54 ^o^C/ null allele | 54 ^o^C | YES |  |
|  |  | R-CTCCGATATTGCCTTTACC |  |  |  |  |  |
| 16 | Sl8-49-FAM | F-CATCTGTTCTTTGAATAGAGG | 52 ^o^C | Incomplete amplification | 52 ^o^C | YES |  |
|  |  | R-ACCCATTCTGGTTCACC |  |  |  |  |  |
| 17 | Sl9-69-FAM | F-TTCCAAACATACCCCTGCC | 54 ^o^C | 54 ^o^C | 54 ^o^C | YES |  |
|  |  | R-ACTAGATGCCACTTGCACC |  |  |  |  |  |
| 18 | Sl10-26-HEX | F-GCTATGTTATATCTATCTTCC | Incomplete amplification | Incomplete amplification | 54 ^o^C /null allele | - |  |
|  |  | R-TTAGGGCATTAATTTGAGTGC |  |  |  |  |  |
| 19 | 3415–416-TAMRA | F-GTTCCTTTCCTTACA | - | - | - | - |  |
|  |  | R-ATCAATGTTTGTCAA |  |  |  |  |  |
| 20 | 4EV1-TAMRA | F-CCCTGCTCCCCATTCTC | 50 ^o^C | - | 50 ^o^C | YES |  |
|  |  | R-ATAAACTCTAATACACTTCCTCCAAC |  |  |  |  |  |

**References**

1. Escorza-Trevino, S.; Archer, F.I.; Rosales, M.; Lang, A.; Dizon, A.E. Genetic differentiation and intraspecific structure of Eastern Tropical Pacific spotted dolphins, Stenella attenuata, revealed by DNA analyses. *Conservation Genetics* **2005**, *6*, 587-600.

2. Farro, A.; Rollo, M.; Silva, J.; Marino, C. Isolation and characterization of microsatellite DNA markers for spinner dolphin (Stenella longirostris). *Conservation Genetics* **2008**, *9*, 1319-1321.

3. Mirimin, L.; Coughlan, J.; Rogan, E.; Cross, T. Tetranucleotide microsatellite loci from the striped dolphin (Stenella coeruleoalba Meyen, 1833). *Molecular ecology notes* **2006**, *6*, 493-495.

4. Faria, D.M.; Steel, D.; Baker, C.S.; da Silva, J.M.; de Meirelles, A.C.O.; Souto, L.R.A.; Siciliano, S.; Barbosa, L.A.; Secchi, E.; Di Tullio, J.C. Mitochondrial diversity and inter-specific phylogeny among dolphins of the genus Stenella in the Southwest Atlantic Ocean. *PLoS One* **2022**, *17*, e0270690.
