# Supplementary material for: Genetic Diversity and Inter‐Specific Phylogeny of Three Sympatric Cetacean Species (Stenella spp.) in Thai Territorial Waters Based on Mitochondrial and Nuclear DNA Markers
Source: Ecol Evol. 2025 Oct 12;15(10):e72322. doi: 10.1002/ece3.72322 (PMC12516012; doi:10.1002/ece3.72322)
Supplement: Supplementary file 4 — Table S3: All sequences and accession numbers used in this study. [file ECE3-15-e72322-s003.docx]

**The genetic diversity and inter-specific phylogeny of three sympatric cetacean species (*Stenella* spp.) in Thai territorial waters based on mitochondrial and nuclear DNA markers**

Promporn Piboon^1^, Janine Brown^2^, Patcharaporn Kaewmong^3^, Kongkiat Kittiwattanawong^4^ Sarisa Klinhom^1^, Toshiaki Yamamoto^5^, and Korakot Nganvongpanit^1,^*

^1^ The School of Veterinary Medicine, Faculty of Veterinary Medicine, Chiang Mai University, Chiang Mai 50100, Thailand.

^2^ Smithsonian Conservation Biology Institute, Center for Species Survival, 1500 Remount Rd, Front Royal, VA, United States.

^3^ Phuket Marine Biological Center, Phuket 83000, Thailand.

^4^ Department of Marine and Coastal Resources, Ratthaprasasanabhakti Building (Building B) The Government Complex, Bangkok 10210, Thailand

^5^ Department of Veterinary Nursing and Technology, Nippon Veterinary and Life Science University, Musashino, Tokyo, Japan

* Correspondence: korakot.n@cmu.ac.th

E-mail:

PP = promporn.piboon@cmu.ac.th

JB= BrownJan@si.edu

PK = marineanimal.vet@gmail.com

KK = kkongkiat@gmail.com

SK= Yui.sarisarisa@gmail.com

TY= tyamamoto@nvlu.ac.jp

KN = korakot.n@cmu.ac.th

**Table S3.** All sequences and accession numbers used in this study.

| **No** | **Species** | **Accession number** | **References** |
| --- | --- | --- | --- |
| 1 | *Stenella attenuata* | GU256406 | Courbis, et al. 2014[1] |
| 2 | *Stenella attenuata* | GQ852569 |  |
| 3 | *Stenella attenuata* | GQ852579 |  |
| 4 | *Stenella attenuata* | GQ852570 |  |
| 5 | *Stenella attenuata* | GU136595 |  |
| 6 | *Stenella attenuata* | GQ852573 |  |
| 7 | *Stenella attenuata* | GQ852568 |  |
| 8 | *Stenella attenuata* | GQ852576 |  |
| 9 | *Stenella attenuata* | GQ852567 |  |
| 10 | *Stenella attenuata* | GQ852571 |  |
| 11 | *Stenella attenuata* | GQ852572 |  |
| 12 | *Stenella attenuata* | GQ852574 |  |
| 13 | *Stenella attenuata* | GQ852578 |  |
| 14 | *Stenella attenuata* | GQ852577 |  |
| 15 | *Stenella attenuata* | GQ852575 |  |
| 16 | *Stenella attenuata* | DQ150139 | Escorza-Trevino, et al. 2005[2] |
| 17 | *Stenella attenuata* | DQ150148 |  |
| 18 | *Stenella attenuata* | DQ150154 |  |
| 19 | *Stenella attenuata* | DQ150174 |  |
| 20 | *Stenella attenuata* | DQ150191 |  |
| 21 | *Stenella attenuata* | DQ150224 |  |
| 22 | *Stenella attenuata* | DQ150227 |  |
| 23 | *Stenella attenuata* | DQ150237 |  |
| 24 | *Stenella attenuata* | DQ150239 |  |
| 25 | *Stenella attenuata* | DQ150242 |  |
| 26 | *Stenella attenuata* | DQ150163 |  |
| 27 | *Stenella attenuata* | DQ150245 |  |
| 28 | *Stenella attenuata* | DQ150151 |  |
| 29 | *Stenella attenuata* | DQ150214 |  |
| 30 | *Stenella attenuata* | DQ150166 |  |
| 31 | *Stenella attenuata* | DQ150150 |  |
| 32 | *Stenella attenuata* | DQ150209 |  |
| 33 | *Stenella attenuata* | DQ150187 |  |
| 34 | *Stenella attenuata* | DQ150161 |  |
| 35 | *Stenella attenuata* | DQ150219 |  |
| 36 | *Stenella attenuata* | DQ150159 |  |
| 37 | *Stenella attenuata* | DQ150157 |  |
| 38 | *Stenella attenuata* | DQ150212 |  |
| 39 | *Stenella attenuata* | DQ150211 |  |
| 40 | *Stenella attenuata* | DQ150155 |  |
| 41 | *Stenella attenuata* | DQ150153 |  |
| 42 | *Stenella attenuata* | DQ150203 |  |
| 43 | *Stenella attenuata* | DQ150149 |  |
| 44 | *Stenella attenuata* | DQ150158 |  |
| 45 | *Stenella attenuata* | DQ150197 |  |
| 46 | *Stenella attenuata* | DQ150196 |  |
| 47 | *Stenella attenuata* | DQ150199 |  |
| 48 | *Stenella attenuata* | DQ150204 |  |
| 49 | *Stenella attenuata* | DQ150152 |  |
| 50 | *Stenella attenuata* | DQ150190 |  |
| 51 | *Stenella attenuata* | DQ150221 |  |
| 52 | *Stenella attenuata* | DQ150207 |  |
| 53 | *Stenella attenuata* | DQ150145 |  |
| 54 | *Stenella attenuata* | DQ150180 |  |
| 55 | *Stenella attenuata* | DQ150195 |  |
| 56 | *Stenella attenuata* | DQ150156 |  |
| 57 | *Stenella attenuata* | DQ150137 |  |
| 58 | *Stenella attenuata* | DQ150138 |  |
| 59 | *Stenella attenuata* | DQ150215 |  |
| 60 | *Stenella attenuata* | DQ150220 |  |
| 61 | *Stenella attenuata* | DQ150160 |  |
| 62 | *Stenella attenuata* | DQ150164 |  |
| 63 | *Stenella attenuata* | DQ150172 |  |
| 64 | *Stenella attenuata* | DQ150181 |  |
| 65 | *Stenella attenuata* | DQ150223 |  |
| 66 | *Stenella attenuata* | DQ150162 |  |
| 67 | *Stenella attenuata* | DQ150147 |  |
| 68 | *Stenella attenuata* | DQ150146 |  |
| 69 | *Stenella attenuata* | DQ150189 |  |
| 70 | *Stenella attenuata* | DQ150141 |  |
| 71 | *Stenella attenuata* | DQ150143 |  |
| 72 | *Stenella attenuata* | DQ150188 |  |
| 73 | *Stenella attenuata* | DQ150142 |  |
| 74 | *Stenella attenuata* | DQ150179 |  |
| 75 | *Stenella attenuata* | DQ150178 |  |
| 76 | *Stenella attenuata* | DQ150177 |  |
| 77 | *Stenella attenuata* | DQ150176 |  |
| 78 | *Stenella attenuata* | DQ150226 |  |
| 79 | *Stenella attenuata* | DQ150182 |  |
| 80 | *Stenella attenuata* | DQ150183 |  |
| 81 | *Stenella attenuata* | DQ150184 |  |
| 82 | *Stenella attenuata* | DQ150185 |  |
| 83 | *Stenella attenuata* | DQ150186 |  |
| 84 | *Stenella attenuata* | DQ150175 |  |
| 85 | *Stenella attenuata* | DQ150144 |  |
| 86 | *Stenella attenuata* | DQ150173 |  |
| 87 | *Stenella attenuata* | DQ150192 |  |
| 88 | *Stenella attenuata* | DQ150193 |  |
| 89 | *Stenella attenuata* | DQ150194 |  |
| 90 | *Stenella attenuata* | DQ150140 |  |
| 91 | *Stenella attenuata* | DQ150135 |  |
| 92 | *Stenella attenuata* | DQ150167 |  |
| 93 | *Stenella attenuata* | DQ150228 |  |
| 94 | *Stenella attenuata* | DQ150244 |  |
| 95 | *Stenella attenuata* | DQ150198 |  |
| 96 | *Stenella attenuata* | DQ150243 |  |
| 97 | *Stenella attenuata* | DQ150200 |  |
| 98 | *Stenella attenuata* | DQ150201 |  |
| 99 | *Stenella attenuata* | DQ150232 |  |
| 100 | *Stenella attenuata* | DQ150202 |  |
| 101 | *Stenella attenuata* | DQ150171 |  |
| 102 | *Stenella attenuata* | DQ150136 |  |
| 103 | *Stenella attenuata* | DQ150205 |  |
| 104 | *Stenella attenuata* | DQ150206 |  |
| 105 | *Stenella attenuata* | DQ150170 |  |
| 106 | *Stenella attenuata* | DQ150208 |  |
| 107 | *Stenella attenuata* | DQ150210 |  |
| 108 | *Stenella attenuata* | DQ150240 |  |
| 109 | *Stenella attenuata* | DQ150169 |  |
| 110 | *Stenella attenuata* | DQ150134 |  |
| 111 | *Stenella attenuata* | DQ150213 |  |
| 112 | *Stenella attenuata* | DQ150168 |  |
| 113 | *Stenella attenuata* | DQ150216 |  |
| 114 | *Stenella attenuata* | DQ150217 |  |
| 115 | *Stenella attenuata* | DQ150218 |  |
| 116 | *Stenella attenuata* | DQ150236 |  |
| 117 | *Stenella attenuata* | DQ150235 |  |
| 118 | *Stenella attenuata* | DQ150222 |  |
| 119 | *Stenella attenuata* | DQ150225 |  |
| 120 | *Stenella attenuata* | DQ150231 |  |
| 121 | *Stenella attenuata* | DQ150165 |  |
| 122 | *Stenella attenuata* | DQ150230 |  |
| 123 | *Stenella attenuata* | DQ150229 |  |
| 124 | *Stenella attenuata* | DQ150233 |  |
| 125 | *Stenella attenuata* | DQ150234 |  |
| 126 | *Stenella attenuata* | DQ150238 |  |
| 127 | *Stenella attenuata* | DQ150241 |  |
| 128 | *Stenella attenuata* | EF438305 | Jayasankar, et al. 2007[3] |
| 129 | *Stenella attenuata* | GQ504129 | Kingston, et al. 2009[4] |
| 130 | *Stenella attenuata* | GQ504128 |  |
| 131 | *Stenella attenuata* | GQ504126 |  |
| 132 | *Stenella attenuata* | GQ504127 |  |
| 133 | *Stenella attenuata* | GQ504124 |  |
| 134 | *Stenella attenuata* | GQ504123 |  |
| 135 | *Stenella attenuata* | GQ504122 |  |
| 136 | *Stenella attenuata* | GQ504121 |  |
| 137 | *Stenella attenuata* | DQ845443 |  |
| 138 | *Stenella attenuata* | DQ845442 |  |
| 139 | *Stenella attenuata* | GQ504120 |  |
| 140 | *Stenella attenuata* | AB610377 |  |
| 141 | *Stenella attenuata* | AB610378 |  |
| 142 | *Stenella attenuata* | KP756629 | Oremus, et al. 2015[5] |
| 143 | *Stenella attenuata* | KP756631 |  |
| 144 | *Stenella attenuata* | KP756632 |  |
| 145 | *Stenella attenuata* | KP756633 |  |
| 146 | *Stenella attenuata* | KP756628 |  |
| 147 | *Stenella attenuata* | KP756630 |  |
| 148 | *Stenella attenuata* | KP756636 |  |
| 149 | *Stenella attenuata* | KP756639 |  |
| 150 | *Stenella attenuata* | KP756640 |  |
| 151 | *Stenella attenuata* | KP756626 |  |
| 152 | *Stenella attenuata* | KP756648 |  |
| 153 | *Stenella attenuata* | KP756627 |  |
| 154 | *Stenella attenuata* | KP756634 |  |
| 155 | *Stenella attenuata* | KP756637 |  |
| 156 | *Stenella attenuata* | KP756638 |  |
| 157 | *Stenella attenuata* | KP756635 |  |
| 158 | *Stenella attenuata* | MZ401227 | This study |
| 159 | *Stenella attenuata* | MZ401210 |  |
| 160 | *Stenella attenuata* | MZ401212 |  |
| 161 | *Stenella attenuata* | MZ401214 |  |
| 162 | *Stenella attenuata* | MZ401215 |  |
| 163 | *Stenella attenuata* | MZ401218 |  |
| 164 | *Stenella attenuata* | MZ401221 |  |
| 165 | *Stenella attenuata* | MZ401226 |  |
| 166 | *Stenella attenuata* | MZ401217 |  |
| 167 | *Stenella attenuata* | MZ401222 |  |
| 168 | *Stenella attenuata* | MZ401223 |  |
| 169 | *Stenella attenuata* | MZ401224 |  |
| 170 | *Stenella attenuata* | MZ401225 |  |
| 171 | *Stenella attenuata* | MZ401230 |  |
| 172 | *Stenella attenuata* | MZ401219 |  |
| 173 | *Stenella attenuata* | MZ401228 |  |
| 174 | *Stenella attenuata* | MZ401229 |  |
| 175 | *Stenella attenuata* | MZ401231 |  |
| 176 | *Stenella attenuata* | MZ401209 |  |
| 177 | *Stenella attenuata* | MZ401216 |  |
| 178 | *Stenella attenuata* | JX414597 | Viricel and Rosel 2021[6] |
| 179 | *Stenella attenuata* | KY963305 | Yusmalinda, et al. 2017[7] |
| 1 | *Stenella coeruleoalba* | OQ736596 | Garrigue, et al. 2023[8] |
| 2 | *Stenella coeruleoalba* | OQ736595 |  |
| 3 | *Stenella coeruleoalba* | OQ736594 |  |
| 4 | *Stenella coeruleoalba* | OQ736593 |  |
| 5 | *Stenella coeruleoalba* | OQ736592 |  |
| 6 | *Stenella coeruleoalba* | OQ736591 |  |
| 7 | *Stenella coeruleoalba* | OQ736590 |  |
| 8 | *Stenella coeruleoalba* | KF258217 | Floridia 2013[9] |
| 9 | *Stenella coeruleoalba* | KF258216 |  |
| 10 | *Stenella coeruleoalba* | KF258215 |  |
| 11 | *Stenella coeruleoalba* | EU079121 | Galov, et al. 2009[10] |
| 12 | *Stenella coeruleoalba* | EU079120 |  |
| 13 | *Stenella coeruleoalba* | EU079119 |  |
| 14 | *Stenella coeruleoalba* | EU079118 |  |
| 15 | *Stenella coeruleoalba* | EU079117 |  |
| 16 | *Stenella coeruleoalba* | EF624063 |  |
| 17 | *Stenella coeruleoalba* | EF624062 |  |
| 18 | *Stenella coeruleoalba* | GQ504160 | Kingston, et al. 2009[4] |
| 19 | *Stenella coeruleoalba* | DQ845441 |  |
| 20 | *Stenella coeruleoalba* | GQ504164 |  |
| 21 | *Stenella coeruleoalba* | GQ504163 |  |
| 22 | *Stenella coeruleoalba* | GQ504162 |  |
| 23 | *Stenella coeruleoalba* | GQ504161 |  |
| 24 | *Stenella coeruleoalba* | GQ504159 |  |
| 25 | *Stenella coeruleoalba* | GQ504158 |  |
| 26 | *Stenella coeruleoalba* | GQ504157 |  |
| 27 | *Stenella coeruleoalba* | GQ504156 |  |
| 28 | *Stenella coeruleoalba* | GQ504154 |  |
| 29 | *Stenella coeruleoalba* | GQ504153 |  |
| 30 | *Stenella coeruleoalba* | GQ504152 |  |
| 31 | *Stenella coeruleoalba* | GQ504149 |  |
| 32 | *Stenella coeruleoalba* | DQ845440 |  |
| 33 | *Stenella coeruleoalba* | AB610381 | Kitamura 2013[11] |
| 34 | *Stenella coeruleoalba* | AB610380 |  |
| 35 | *Stenella coeruleoalba* | ON959825 | Linguiti, et al. 2021[12] |
| 36 | *Stenella coeruleoalba* | ON959820 |  |
| 37 | *Stenella coeruleoalba* | ON959826 |  |
| 38 | *Stenella coeruleoalba* | ON959824 |  |
| 39 | *Stenella coeruleoalba* | ON959823 |  |
| 40 | *Stenella coeruleoalba* | ON959822 |  |
| 41 | *Stenella coeruleoalba* | ON959821 |  |
| 42 | *Stenella coeruleoalba* | ON959830 |  |
| 43 | *Stenella coeruleoalba* | ON959827 |  |
| 44 | *Stenella coeruleoalba* | ON959831 |  |
| 45 | *Stenella coeruleoalba* | ON959829 |  |
| 46 | *Stenella coeruleoalba* | ON959828 |  |
| 47 | *Stenella coeruleoalba* | AM498725 | Mace, et al. 2006[13] |
| 48 | *Stenella coeruleoalba* | AM498724 |  |
| 49 | *Stenella coeruleoalba* | AM498723 |  |
| 50 | *Stenella coeruleoalba* | AM498722 |  |
| 51 | *Stenella coeruleoalba* | AM498721 |  |
| 52 | *Stenella coeruleoalba* | AM498720 |  |
| 53 | *Stenella coeruleoalba* | AM498719 |  |
| 54 | *Stenella coeruleoalba* | AM498718 |  |
| 55 | *Stenella coeruleoalba* | AM498717 |  |
| 56 | *Stenella coeruleoalba* | AM498716 |  |
| 57 | *Stenella coeruleoalba* | AM498715 |  |
| 58 | *Stenella coeruleoalba* | AM498714 |  |
| 59 | *Stenella coeruleoalba* | AM498713 |  |
| 60 | *Stenella coeruleoalba* | AM498712 |  |
| 61 | *Stenella coeruleoalba* | AM498711 |  |
| 62 | *Stenella coeruleoalba* | AM498710 |  |
| 63 | *Stenella coeruleoalba* | AM498709 |  |
| 64 | *Stenella coeruleoalba* | AM498708 |  |
| 65 | *Stenella coeruleoalba* | AM498707 |  |
| 66 | *Stenella coeruleoalba* | AM498686 |  |
| 67 | *Stenella coeruleoalba* | AM498739 |  |
| 68 | *Stenella coeruleoalba* | AM498692 |  |
| 69 | *Stenella coeruleoalba* | AM498689 |  |
| 70 | *Stenella coeruleoalba* | AM498681 |  |
| 71 | *Stenella coeruleoalba* | AM498690 |  |
| 72 | *Stenella coeruleoalba* | AM498736 |  |
| 73 | *Stenella coeruleoalba* | AM498696 |  |
| 74 | *Stenella coeruleoalba* | AM498684 |  |
| 75 | *Stenella coeruleoalba* | AM498674 |  |
| 76 | *Stenella coeruleoalba* | AM498735 |  |
| 77 | *Stenella coeruleoalba* | AM498734 |  |
| 78 | *Stenella coeruleoalba* | AM498733 |  |
| 79 | *Stenella coeruleoalba* | AM498732 |  |
| 80 | *Stenella coeruleoalba* | AM498731 |  |
| 81 | *Stenella coeruleoalba* | AM498730 |  |
| 82 | *Stenella coeruleoalba* | AM498727 |  |
| 83 | *Stenella coeruleoalba* | AM498726 |  |
| 84 | *Stenella coeruleoalba* | AM498700 |  |
| 85 | *Stenella coeruleoalba* | AM498685 |  |
| 86 | *Stenella coeruleoalba* | AM498673 |  |
| 87 | *Stenella coeruleoalba* | AM498668 |  |
| 88 | *Stenella coeruleoalba* | AM498738 |  |
| 89 | *Stenella coeruleoalba* | AM498679 |  |
| 90 | *Stenella coeruleoalba* | AM498729 |  |
| 91 | *Stenella coeruleoalba* | AM498728 |  |
| 92 | *Stenella coeruleoalba* | AM498672 |  |
| 93 | *Stenella coeruleoalba* | AM498737 |  |
| 94 | *Stenella coeruleoalba* | AM498680 |  |
| 95 | *Stenella coeruleoalba* | AM498676 |  |
| 96 | *Stenella coeruleoalba* | AM498682 |  |
| 97 | *Stenella coeruleoalba* | AM498678 |  |
| 98 | *Stenella coeruleoalba* | AM498699 |  |
| 99 | *Stenella coeruleoalba* | AM498698 |  |
| 100 | *Stenella coeruleoalba* | AM498697 |  |
| 101 | *Stenella coeruleoalba* | AM498695 |  |
| 102 | *Stenella coeruleoalba* | AM498694 |  |
| 103 | *Stenella coeruleoalba* | AM498691 |  |
| 104 | *Stenella coeruleoalba* | AM498688 |  |
| 105 | *Stenella coeruleoalba* | AM498687 |  |
| 106 | *Stenella coeruleoalba* | AM498683 |  |
| 107 | *Stenella coeruleoalba* | AM498677 |  |
| 108 | *Stenella coeruleoalba* | AM498675 |  |
| 109 | *Stenella coeruleoalba* | AM498671 |  |
| 110 | *Stenella coeruleoalba* | AM498670 |  |
| 111 | *Stenella coeruleoalba* | AM498669 |  |
| 112 | *Stenella coeruleoalba* | AM498667 |  |
| 113 | *Stenella coeruleoalba* | AM498693 |  |
| 114 | *Stenella coeruleoalba* | AM498740 |  |
| 115 | *Stenella coeruleoalba* | AM498706 |  |
| 116 | *Stenella coeruleoalba* | AM498705 |  |
| 117 | *Stenella coeruleoalba* | AM498704 |  |
| 118 | *Stenella coeruleoalba* | AM498703 |  |
| 119 | *Stenella coeruleoalba* | AM498702 |  |
| 120 | *Stenella coeruleoalba* | AM498701 |  |
| 121 | *Stenella coeruleoalba* | AY046549 | Guang, et al. 2002[14] |
| 122 | *Stenella coeruleoalba* | AY046547 |  |
| 123 | *Stenella coeruleoalba* | AY046544 |  |
| 124 | *Stenella coeruleoalba* | AY046540 |  |
| 125 | *Stenella coeruleoalba* | AY046542 |  |
| 126 | *Stenella coeruleoalba* | AY046543 |  |
| 127 | *Stenella coeruleoalba* | AY046541 |  |
| 128 | *Stenella coeruleoalba* | AY046539 |  |
| 129 | *Stenella coeruleoalba* | AY046546 |  |
| 130 | *Stenella coeruleoalba* | AY046548 |  |
| 131 | *Stenella coeruleoalba* | AY046545 |  |
| 132 | *Stenella coeruleoalba* | MZ401298 | This study |
| 133 | *Stenella coeruleoalba* | MZ401312 |  |
| 134 | *Stenella coeruleoalba* | MZ401307 |  |
| 135 | *Stenella coeruleoalba* | MZ401317 |  |
| 136 | *Stenella coeruleoalba* | MZ401310 |  |
| 137 | *Stenella coeruleoalba* | MZ401309 |  |
| 138 | *Stenella coeruleoalba* | MZ401311 |  |
| 139 | *Stenella coeruleoalba* | MZ401313 |  |
| 140 | *Stenella coeruleoalba* | MZ401314 |  |
| 141 | *Stenella coeruleoalba* | MZ401316 |  |
| 142 | *Stenella coeruleoalba* | MZ401318 |  |
| 143 | *Stenella coeruleoalba* | MZ401319 |  |
| 144 | *Stenella coeruleoalba* | MZ401321 |  |
| 145 | *Stenella coeruleoalba* | MZ401320 |  |
| 146 | *Stenella coeruleoalba* | MZ401327 |  |
| 147 | *Stenella coeruleoalba* | MZ401322 |  |
| 148 | *Stenella coeruleoalba* | MZ401299 |  |
| 149 | *Stenella coeruleoalba* | MZ401323 |  |
| 150 | *Stenella coeruleoalba* | MZ401324 |  |
| 151 | *Stenella coeruleoalba* | MZ401328 |  |
| 152 | *Stenella coeruleoalba* | MZ401331 |  |
| 153 | *Stenella coeruleoalba* | MZ401329 |  |
| 154 | *Stenella coeruleoalba* | MZ401330 |  |
| 155 | *Stenella coeruleoalba* | MZ401332 |  |
| 156 | *Stenella coeruleoalba* | MZ401308 |  |
| 157 | *Stenella coeruleoalba* | MZ401300 |  |
| 158 | *Stenella coeruleoalba* | MZ401301 |  |
| 159 | *Stenella coeruleoalba* | MZ401325 |  |
| 160 | *Stenella coeruleoalba* | MZ401326 |  |
| 161 | *Stenella coeruleoalba* | MZ401302 |  |
| 162 | *Stenella coeruleoalba* | MZ401303 |  |
| 163 | *Stenella coeruleoalba* | MZ401304 |  |
| 164 | *Stenella coeruleoalba* | MZ401315 |  |
| 165 | *Stenella coeruleoalba* | MZ401305 |  |
| 166 | *Stenella coeruleoalba* | MZ401306 |  |
| 167 | *Stenella coeruleoalba* | MZ401333 |  |
| 1 | *Stenella longirostris* | MK184992 | Faria, et al. 2022[15] |
| 2 | *Stenella longirostris* | MK184993 |  |
| 3 | *Stenella longirostris* | MK184994 |  |
| 4 | *Stenella longirostris* | MK184995 |  |
| 5 | *Stenella longirostris* | MK184996 |  |
| 6 | *Stenella longirostris* | MK184997 |  |
| 7 | *Stenella longirostris* | MK184998 |  |
| 8 | *Stenella longirostris* | MK184999 |  |
| 9 | *Stenella longirostris* | MK185000 |  |
| 10 | *Stenella longirostris* | MK185001 |  |
| 11 | *Stenella longirostris* | MK185002 |  |
| 12 | *Stenella longirostris* | KX905105 | Viricel, et al. 2016[16] |
| 13 | *Stenella longirostris* | KX905106 |  |
| 14 | *Stenella longirostris* | KX905107 |  |
| 15 | *Stenella longirostris* | KX905108 |  |
| 16 | *Stenella longirostris* | KX905109 |  |
| 17 | *Stenella longirostris* | KX905110 |  |
| 18 | *Stenella longirostris* | KX905111 |  |
| 19 | *Stenella longirostris* | KX905112 |  |
| 20 | *Stenella longirostris* | KX905113 |  |
| 21 | *Stenella longirostris* | KX905114 |  |
| 22 | *Stenella longirostris* | KX905115 |  |
| 23 | *Stenella longirostris* | KX905116 |  |
| 24 | *Stenella longirostris* | KX905117 |  |
| 25 | *Stenella longirostris* | KX905118 |  |
| 26 | *Stenella longirostris* | KX905119 |  |
| 27 | *Stenella longirostris* | KX905120 |  |
| 28 | *Stenella longirostris* | KX905121 |  |
| 29 | *Stenella longirostris* | KX905122 |  |
| 30 | *Stenella longirostris* | KX905123 |  |
| 31 | *Stenella longirostris* | KX905124 |  |
| 32 | *Stenella longirostris* | KX905125 |  |
| 33 | *Stenella longirostris* | KX905126 |  |
| 34 | *Stenella longirostris* | KX905127 |  |
| 35 | *Stenella longirostris* | KX905128 |  |
| 36 | *Stenella longirostris* | KX905129 |  |
| 37 | *Stenella longirostris* | KX905130 |  |
| 38 | *Stenella longirostris* | KX905131 |  |
| 39 | *Stenella longirostris* | KX905132 |  |
| 40 | *Stenella longirostris* | KY457781 | Martien, et al. 2014[17] |
| 41 | *Stenella longirostris* | KY457782 |  |
| 42 | *Stenella longirostris* | KY457783 |  |
| 43 | *Stenella longirostris* | KY457784 |  |
| 44 | *Stenella longirostris* | KY457785 |  |
| 45 | *Stenella longirostris* | KY457786 |  |
| 46 | *Stenella longirostris* | KY457787 |  |
| 47 | *Stenella longirostris* | KY457788 |  |
| 48 | *Stenella longirostris* | KY457789 |  |
| 49 | *Stenella longirostris* | KY457790 |  |
| 50 | *Stenella longirostris* | KY457791 |  |
| 51 | *Stenella longirostris* | KY457792 |  |
| 52 | *Stenella longirostris* | KY457793 |  |
| 53 | *Stenella longirostris* | KY457794 |  |
| 54 | *Stenella longirostris* | KY457795 |  |
| 55 | *Stenella longirostris* | KY457796 |  |
| 56 | *Stenella longirostris* | KY457797 |  |
| 57 | *Stenella longirostris* | KY457798 |  |
| 58 | *Stenella longirostris* | KY457799 |  |
| 59 | *Stenella longirostris* | KY457800 |  |
| 60 | *Stenella longirostris* | KY457801 |  |
| 61 | *Stenella longirostris* | KY457802 |  |
| 62 | *Stenella longirostris* | KY457803 |  |
| 63 | *Stenella longirostris* | KY457804 |  |
| 64 | *Stenella longirostris* | KP756643 | Oremus, et al. 2015[5] |
| 65 | *Stenella longirostris* | KP756644 |  |
| 66 | *Stenella longirostris* | GU253256 | Andrews, et al. 2010[18] |
| 67 | *Stenella longirostris* | GU253257 |  |
| 68 | *Stenella longirostris* | GU253258 |  |
| 69 | *Stenella longirostris* | GU253259 |  |
| 70 | *Stenella longirostris* | GU253260 |  |
| 71 | *Stenella longirostris* | GU253261 |  |
| 72 | *Stenella longirostris* | GU253262 |  |
| 73 | *Stenella longirostris* | GU253263 |  |
| 74 | *Stenella longirostris* | GU253264 |  |
| 75 | *Stenella longirostris* | GU253265 |  |
| 76 | *Stenella longirostris* | GU253266 |  |
| 77 | *Stenella longirostris* | GU253267 |  |
| 78 | *Stenella longirostris* | GU253268 |  |
| 79 | *Stenella longirostris* | GU253269 |  |
| 80 | *Stenella longirostris* | GU253270 |  |
| 81 | *Stenella longirostris* | GU253271 |  |
| 82 | *Stenella longirostris* | GU253272 |  |
| 83 | *Stenella longirostris* | GU253273 |  |
| 84 | *Stenella longirostris* | GU253274 |  |
| 85 | *Stenella longirostris* | GU253275 |  |
| 86 | *Stenella longirostris* | GU253276 |  |
| 87 | *Stenella longirostris* | GU253277 |  |
| 88 | *Stenella longirostris* | GU253278 |  |
| 89 | *Stenella longirostris* | GU253279 |  |
| 90 | *Stenella longirostris* | GU253280 |  |
| 91 | *Stenella longirostris* | GU253281 |  |
| 92 | *Stenella longirostris* | GU253282 |  |
| 93 | *Stenella longirostris* | GU253283 |  |
| 94 | *Stenella longirostris* | GU253284 |  |
| 95 | *Stenella longirostris* | EF558737 | Oremus, et al. 2007[19] |
| 96 | *Stenella longirostris* | EF558738 |  |
| 97 | *Stenella longirostris* | EF558739 |  |
| 98 | *Stenella longirostris* | EF558740 |  |
| 99 | *Stenella longirostris* | EF558741 |  |
| 100 | *Stenella longirostris* | EF558742 |  |
| 101 | *Stenella longirostris* | EF558743 |  |
| 102 | *Stenella longirostris* | EF558744 |  |
| 103 | *Stenella longirostris* | EF558745 |  |
| 104 | *Stenella longirostris* | EF558746 |  |
| 105 | *Stenella longirostris* | EF558747 |  |
| 106 | *Stenella longirostris* | EF558748 |  |
| 107 | *Stenella longirostris* | EF558749 |  |
| 108 | *Stenella longirostris* | EF558750 |  |
| 109 | *Stenella longirostris* | EF558751 |  |
| 110 | *Stenella longirostris* | EF558752 |  |
| 111 | *Stenella longirostris* | EF558753 |  |
| 112 | *Stenella longirostris* | EF558754 |  |
| 113 | *Stenella longirostris* | EF558755 |  |
| 114 | *Stenella longirostris* | EF558756 |  |
| 115 | *Stenella longirostris* | EF558757 |  |
| 116 | *Stenella longirostris* | EF558758 |  |
| 117 | *Stenella longirostris* | EF558759 |  |
| 118 | *Stenella longirostris* | EF558760 |  |
| 119 | *Stenella longirostris* | EF558761 |  |
| 120 | *Stenella longirostris* | EF558762 |  |
| 121 | *Stenella longirostris* | EF558763 |  |
| 122 | *Stenella longirostris* | EF558764 |  |
| 123 | *Stenella longirostris* | EF558765 |  |
| 124 | *Stenella longirostris* | EF558766 |  |
| 125 | *Stenella longirostris* | EF558767 |  |
| 126 | *Stenella longirostris* | KC160997 | Andrews, et al. 2013[20] |
| 127 | *Stenella longirostris* | KC160998 |  |
| 128 | *Stenella longirostris* | KC160999 |  |
| 129 | *Stenella longirostris* | KC161018 |  |
| 130 | *Stenella longirostris* | KC161021 |  |
| 131 | *Stenella longirostris* | KC161037 |  |
| 132 | *Stenella longirostris* | KC161038 |  |
| 133 | *Stenella longirostris* | KC161039 |  |
| 134 | *Stenella longirostris* | KC161040 |  |
| 135 | *Stenella longirostris* | KC161041 |  |
| 136 | *Stenella longirostris* | KC161042 |  |
| 137 | *Stenella longirostris* | KC161043 |  |
| 138 | *Stenella longirostris* | KC161044 |  |
| 139 | *Stenella longirostris* | KC161045 |  |
| 140 | *Stenella longirostris* | KC161046 |  |
| 141 | *Stenella longirostris* | KC161047 |  |
| 142 | *Stenella longirostris* | KC161048 |  |
| 143 | *Stenella longirostris* | KC161049 |  |
| 144 | *Stenella longirostris* | KC161050 |  |
| 145 | *Stenella longirostris* | KC161051 |  |
| 146 | *Stenella longirostris* | KC161052 |  |
| 147 | *Stenella longirostris* | KC161053 |  |
| 148 | *Stenella longirostris* | KC161054 |  |
| 149 | *Stenella longirostris* | KC161055 |  |
| 150 | *Stenella longirostris* | KC161056 |  |
| 151 | *Stenella longirostris* | KC161057 |  |
| 152 | *Stenella longirostris* | KC161058 |  |
| 153 | *Stenella longirostris* | KC161059 |  |
| 154 | *Stenella longirostris* | KC161060 |  |
| 155 | *Stenella longirostris* | KC161061 |  |
| 156 | *Stenella longirostris* | KC161062 |  |
| 157 | *Stenella longirostris* | KC161063 |  |
| 158 | *Stenella longirostris* | KC161064 |  |
| 159 | *Stenella longirostris* | KC161065 |  |
| 160 | *Stenella longirostris* | KC161066 |  |
| 161 | *Stenella longirostris* | KC161067 |  |
| 162 | *Stenella longirostris* | KC161068 |  |
| 163 | *Stenella longirostris* | KC161069 |  |
| 164 | *Stenella longirostris* | KC161070 |  |
| 165 | *Stenella longirostris* | KC161071 |  |
| 166 | *Stenella longirostris* | KC161072 |  |
| 167 | *Stenella longirostris* | KC161073 |  |
| 168 | *Stenella longirostris* | KC161074 |  |
| 169 | *Stenella longirostris* | KC161075 |  |
| 170 | *Stenella longirostris* | KC161076 |  |
| 171 | *Stenella longirostris* | KC161077 |  |
| 172 | *Stenella longirostris* | KC161096 |  |
| 173 | *Stenella longirostris* | KC161097 |  |
| 174 | *Stenella longirostris* | KC161098 |  |
| 175 | *Stenella longirostris* | KC161113 |  |
| 176 | *Stenella longirostris* | KC161114 |  |
| 177 | *Stenella longirostris* | KC161115 |  |
| 178 | *Stenella longirostris* | KC161116 |  |
| 179 | *Stenella longirostris* | KC161117 |  |
| 180 | *Stenella longirostris* | KC161118 |  |
| 181 | *Stenella longirostris* | KC161119 |  |
| 182 | *Stenella longirostris* | KC161120 |  |
| 183 | *Stenella longirostris* | KC161121 |  |
| 184 | *Stenella longirostris* | KC161122 |  |
| 185 | *Stenella longirostris* | KC161123 |  |
| 186 | *Stenella longirostris* | KC161124 |  |
| 187 | *Stenella longirostris* | KC161125 |  |
| 188 | *Stenella longirostris* | MZ401245 | This study |
| 189 | *Stenella longirostris* | MZ401246 |  |
| 190 | *Stenella longirostris* | MZ401247 |  |
| 191 | *Stenella longirostris* | MZ401248 |  |
| 192 | *Stenella longirostris* | MZ401250 |  |
| 193 | *Stenella longirostris* | MZ401251 |  |
| 194 | *Stenella longirostris* | MZ401252 |  |
| 195 | *Stenella longirostris* | MZ401253 |  |
| 196 | *Stenella longirostris* | MZ401258 |  |
| 197 | *Stenella longirostris* | MZ401261 |  |
| 198 | *Stenella longirostris* | MZ401262 |  |
| 199 | *Stenella longirostris* | MZ401263 |  |
| 200 | *Stenella longirostris* | MZ401264 |  |
| 201 | *Stenella longirostris* | MZ401265 |  |
| 202 | *Stenella longirostris* | MZ401266 |  |
| 203 | *Stenella longirostris* | MZ401267 |  |
| 204 | *Stenella longirostris* | MZ401268 |  |
| 205 | *Stenella longirostris* | MZ401269 |  |
| 206 | *Stenella longirostris* | MZ401270 |  |
| 207 | *Stenella longirostris* | MZ401272 |  |
| 208 | *Stenella longirostris* | MZ401273 |  |
| 209 | *Stenella longirostris* | MZ401274 |  |
| 210 | *Stenella longirostris* | MZ401275 |  |
| 211 | *Stenella longirostris* | MZ401276 |  |
| 212 | *Stenella longirostris* | MZ401277 |  |
| 213 | *Stenella longirostris* | MZ401278 |  |
| 214 | *Stenella longirostris* | MZ401280 |  |
| 215 | *Stenella longirostris* | MZ401281 |  |
| 216 | *Stenella longirostris* | MZ401282 |  |
| 217 | *Stenella longirostris* | MZ401284 |  |
| 218 | *Stenella longirostris* | MZ401285 |  |
| 219 | *Stenella longirostris* | MZ401286 |  |
| 220 | *Stenella longirostris* | MZ401287 |  |
| 221 | *Stenella longirostris* | MZ401288 |  |
| 222 | *Stenella longirostris* | MZ401289 |  |
| 223 | *Stenella longirostris* | MZ401290 |  |
| 224 | *Stenella longirostris* | MZ401292 |  |
| 225 | *Stenella longirostris* | MZ401293 |  |
| 226 | *Stenella longirostris* | MZ401294 |  |
| 227 | *Stenella longirostris* | MZ401295 |  |
| 228 | *Stenella longirostris* | MZ401296 |  |
| 229 | *Stenella longirostris* | MZ401297 |  |

**References**

1. Courbis, S.; Baird, R.W.; Cipriano, F.; Duffield, D. Multiple populations of pantropical spotted dolphins in Hawaiian waters. *Journal of Heredity* **2014**, *105*, 627-641.

2. Escorza-Trevino, S.; Archer, F.I.; Rosales, M.; Lang, A.; Dizon, A.E. Genetic differentiation and intraspecific structure of Eastern Tropical Pacific spotted dolphins, Stenella attenuata, revealed by DNA analyses. *Conservation Genetics* **2005**, *6*, 587-600.

3. National Center for Biotechnology Information. Stenella attenuata isolate CH5 mitochondrial control region. **2007**. EF438305. Available online: *accessed on*.

4. Kingston, S.E.; Adams, L.D.; Rosel, P.E. Testing mitochondrial sequences and anonymous nuclear markers for phylogeny reconstruction in a rapidly radiating group: molecular systematics of the Delphininae (Cetacea: Odontoceti: Delphinidae). *BMC Evolutionary Biology* **2009**, *9*, 1-19.

5. Oremus, M.; Leqata, J.; Baker, C.S. Resumption of traditional drive hunting of dolphins in the Solomon Islands in 2013. *Royal Society Open Science* **2015**, *2*, 140524.

6. National Center for Biotechnology Information. Hybridization and introgression in spotted dolphins. **2021**. JX414597. Available online: *accessed on*.

7. Yusmalinda, N.L.A.; Anggoro, A.W.; Suhendro, D.M.; Ratha, I.M.J.; Suprapti, D.; Kreb, D.; Cahyani, N.K.D. Species identification of stranded cetaceans in Indonesia revealed by molecular technique. *Jurnal Ilmu dan Teknologi Kelautan Tropis* **2017**, *9*, 465-474.

8. Garrigue, C.; Derville, S.; Bonneville, C.; Brisset, M.; Bustamante, P.; Cleguer, C.; Clua, E.E.; Dabin, W.; Fiat, S.; Justine, J.-L. Marine mammal strandings recorded in New Caledonia, South West Pacific Ocean, 1877 to 2022. *Pacific Conservation Biology* **2023**, *30*, NULL-NULL.

9. National Center for Biotechnology Information. Stenella coeruleoalba isolate STCSM4 D-loop, partial sequence; mitochondrial. **2013**. KF258217. Available online: *accessed on*.

10. Galov, A.; Lauc, G.; Nikolić, N.; Šatović, Z.; Gomerčić, T.; Gomerčić, M.Đ.; Kocijan, I.; Šeol, B.; Gomerčić, H. Records and genetic diversity of striped dolphins (*Stenella coeruleoalba*) from the Croatian coast of the Adriatic Sea. *Marine Biodiversity Records* **2009**, *2*, e98.

11. National Center for Biotechnology Information. Molecular genetic studies on the Odontoceti. **2013**. AB610380-AB610381. Available online: *accessed on*.

12. Linguiti, G.; Fanizza, C.; Ciani, E.; Bellomo, S.; Cipriano, G.; Santacesaria, F.C.; Ciccarese, S.; Antonacci, R.; Carlucci, R. Assessment of genetic diversity of the striped dolphin population in the Gulf of Taranto (Northern Ionian Sea, Central Mediterranean Sea). In Proceedings of the 2021 International Workshop on Metrology for the Sea; Learning to Measure Sea Health Parameters (MetroSea), 2021; pp. 144-147.

13. Mace, M.; Bourret, V.; Crouau-Roy, B. Polyphyly in the Mediterranean striped dolphin (Stenella coeruleoalba) and the Messinian Salinity Crisis. *Manuscrito enviado a Molecular Ecology* **2006**.

14. Guang, Y.; Wenhua, R.; Minghua, N.; Kaiya, Z. Variability of the complete mitochondrial control region of striped dolphins (Stenella coeruleualba). *Dong wu xue bao.[Acta Zoologica Sinica]* **2002**, *48*, 131-134.

15. Faria, D.M.; Steel, D.; Baker, C.S.; da Silva, J.M.; de Meirelles, A.C.O.; Souto, L.R.A.; Siciliano, S.; Barbosa, L.A.; Secchi, E.; Di Tullio, J.C. Mitochondrial diversity and inter-specific phylogeny among dolphins of the genus Stenella in the Southwest Atlantic Ocean. *PLoS One* **2022**, *17*, e0270690.

16. Viricel, A.; Simon-Bouhet, B.; Ceyrac, L.; Dulau-Drouot, V.; Berggren, P.; Amir, O.A.; Jiddawi, N.S.; Mongin, P.; Kiszka, J.J. Habitat availability and geographic isolation as potential drivers of population structure in an oceanic dolphin in the Southwest Indian Ocean. *Marine biology* **2016**, *163*, 1-12.

17. Martien, K.K.; Hill, M.C.; Van Cise, A.M.; Robertson, K.M.; Woodman, S.M.; Dolar, L.; Pease, V.L.; Oleson, E.M. Genetic diversity and population structure in four species of cetaceans around the Mariana Islands. **2014**.

18. Andrews, K.R.; Karczmarski, L.; Au, W.W.; Rickards, S.H.; Vanderlip, C.A.; Bowen, B.W.; Gordon Grau, E.; Toonen, R.J. Rolling stones and stable homes: social structure, habitat diversity and population genetics of the Hawaiian spinner dolphin (Stenella longirostris). *Molecular Ecology* **2010**, *19*, 732-748.

19. Oremus, M.; Poole, M.M.; Steel, D.; Baker, C.S. Isolation and interchange among insular spinner dolphin communities in the South Pacific revealed by individual identification and genetic diversity. *Marine Ecology Progress Series* **2007**, *336*, 275-289.

20. Andrews, K.R.; Perrin, W.F.; Oremus, M.; Karczmarski, L.; Bowen, B.W.; Puritz, J.B.; Toonen, R.J. The evolving male: spinner dolphin (S tenella longirostris) ecotypes are divergent at Y chromosome but not mt DNA or autosomal markers. *Molecular Ecology* **2013**, *22*, 2408-2423.
