# Supplementary material for: Genetic Diversity and Inter‐Specific Phylogeny of Three Sympatric Cetacean Species (Stenella spp.) in Thai Territorial Waters Based on Mitochondrial and Nuclear DNA Markers
Source: Ecol Evol. 2025 Oct 12;15(10):e72322. doi: 10.1002/ece3.72322 (PMC12516012; doi:10.1002/ece3.72322)
Supplement: Supplementary file 5 — Table S4: The detail of microsatellite loci Stenella attenuata used in this study. [file ECE3-15-e72322-s004.docx]

**The genetic diversity and inter-specific phylogeny of three sympatric cetacean species (*Stenella* spp.) in Thai territorial waters based on mitochondrial and nuclear DNA markers**

Promporn Piboon^1^, Janine Brown^2^, Patcharaporn Kaewmong^3^, Kongkiat Kittiwattanawong^4^ Sarisa Klinhom^1^, Toshiaki Yamamoto^5^, and Korakot Nganvongpanit^1,^*

^1^ The School of Veterinary Medicine, Faculty of Veterinary Medicine, Chiang Mai University, Chiang Mai 50100, Thailand.

^2^ Smithsonian Conservation Biology Institute, Center for Species Survival, 1500 Remount Rd, Front Royal, VA, United States.

^3^ Phuket Marine Biological Center, Phuket 83000, Thailand.

^4^ Department of Marine and Coastal Resources, Ratthaprasasanabhakti Building (Building B) The Government Complex, Bangkok 10210, Thailand

^5^ Department of Veterinary Nursing and Technology, Nippon Veterinary and Life Science University, Musashino, Tokyo, Japan

* Correspondence: korakot.n@cmu.ac.th

E-mail:

PP = promporn.piboon@cmu.ac.th

JB= BrownJan@si.edu

PK = marineanimal.vet@gmail.com

KK = kkongkiat@gmail.com

SK= Yui.sarisarisa@gmail.com

TY= tyamamoto@nvlu.ac.jp

KN = korakot.n@cmu.ac.th

**Table S4.** The detail of microsatellite loci *Stenella attenuata* used in this study

| Locus | N | Na | AR | Ho | He | Fis | PIC | PID | PIDsibs | P(HWE) |
| --- | --- | --- | --- | --- | --- | --- | --- | --- | --- | --- |
| Sl9-69-FAM | 14 | 8 | 7.26 | 0.929 | 0.847 | -0.101 | 0.793 | 0.0575 | 0.3562 | 0.895 |
| Slo4-FAM | 14 | 5 | 4.43 | 0.714 | 0.651 | -0.102 | 0.576 | 0.1907 | 0.4839 | 0.597 |
| Sl8-49-FAM | 15 | 11 | 9.56 | 0.800 | 0.901 | 0.116 | 0.858 | 0.0292 | 0.3218 | 0.352 |
| EV104-HEX | 11 | 3 | 3.00 | 0.545 | 0.589 | 0.077 | 0.476 | 0.2776 | 0.5384 | 0.326 |
| Sl1-25-TAMRA | 11 | 9 | 8.63 | 0.727 | 0.870 | 0.171 | 0.811 | 0.0487 | 0.3469 | 0.093 |
| Sco66-TAMRA | 10 | 3 | 3.00 | 0.800 | 0.595 | -0.371 | 0.482 | 0.2718 | 0.5355 | 0.433 |
| EV1-TAMRA | 13 | 8 | 7.21 | 0.692 | 0.809 | 0.150 | 0.749 | 0.0786 | 0.3806 | 0.222 |
| Sco28-ROX | 15 | 3 | 2.64 | 0.133 | 0.246 | 0.467 | 0.221 | 0.5977 | 0.7805 | 0.205 |
| Mean | 12.88 | 6.25 | 5.72 | 0.668 | 0.689 | 0.051 | 0.621 |  |  |  |
| SD | 1.96 | 3.15 | 2.77 | 0.242 | 0.218 | 0.247 | 0.221 |  |  |  |
