# Supplementary material for: Genetic Diversity and Inter‐Specific Phylogeny of Three Sympatric Cetacean Species (Stenella spp.) in Thai Territorial Waters Based on Mitochondrial and Nuclear DNA Markers
Source: Ecol Evol. 2025 Oct 12;15(10):e72322. doi: 10.1002/ece3.72322 (PMC12516012; doi:10.1002/ece3.72322)
Supplement: Supplementary file 6 — Table S5: The detail of microsatellite loci Stenella coeruleoalba used in this study. [file ECE3-15-e72322-s005.docx]

**The genetic diversity and inter-specific phylogeny of three sympatric cetacean species (*Stenella* spp.) in Thai territorial waters based on mitochondrial and nuclear DNA markers**

Promporn Piboon^1^, Janine Brown^2^, Patcharaporn Kaewmong^3^, Kongkiat Kittiwattanawong^4^ Sarisa Klinhom^1^, Toshiaki Yamamoto^5^, and Korakot Nganvongpanit^1,^*

^1^ The School of Veterinary Medicine, Faculty of Veterinary Medicine, Chiang Mai University, Chiang Mai 50100, Thailand.

^2^ Smithsonian Conservation Biology Institute, Center for Species Survival, 1500 Remount Rd, Front Royal, VA, United States.

^3^ Phuket Marine Biological Center, Phuket 83000, Thailand.

^4^ Department of Marine and Coastal Resources, Ratthaprasasanabhakti Building (Building B) The Government Complex, Bangkok 10210, Thailand

^5^ Department of Veterinary Nursing and Technology, Nippon Veterinary and Life Science University, Musashino, Tokyo, Japan

* Correspondence: korakot.n@cmu.ac.th

E-mail:

PP = promporn.piboon@cmu.ac.th

JB= BrownJan@si.edu

PK = marineanimal.vet@gmail.com

KK = kkongkiat@gmail.com

SK= Yui.sarisarisa@gmail.com

TY= tyamamoto@nvlu.ac.jp

KN = korakot.n@cmu.ac.th

**Table S5.** The detail of microsatellite loci *Stenella coeruleoalba* used in this study

| Locus | N | Na | AR | Ho | He | Fis | PIC | PID | PIDsibs | P(HWE) |
| --- | --- | --- | --- | --- | --- | --- | --- | --- | --- | --- |
| EV104-HEX | 33 | 9 | 8.21 | 0.788 | 0.769 | -0.025 | 0.722 | 0.0944 | 0.3950 | 0.293 |
| Sco11-HEX | 30 | 11 | 10.14 | 0.733 | 0.718 | -0.022 | 0.683 | 0.1096 | 0.4243 | 0.539 |
| Sco66-TAMRA | 28 | 10 | 9.87 | 0.821 | 0.873 | 0.060 | 0.841 | 0.0365 | 0.3306 | 0.136 |
| Sl9-69-FAM | 33 | 8 | 7.74 | 0.848 | 0.825 | -0.029 | 0.789 | 0.0585 | 0.3585 | 0.253 |
| Sl015-HEX | 33 | 8 | 7.26 | 0.848 | 0.745 | -0.141 | 0.695 | 0.1096 | 0.4106 | 0.416 |
| Sl09-HEX | 25 | 3 | 3.00 | 0.200 | 0.189 | -0.062 | 0.176 | 0.6730 | 0.8258 | 1.000 |
| Sco65-ROX | 29 | 4 | 4.00 | 0.414 | 0.479 | 0.137 | 0.440 | 0.3112 | 0.5927 | 0.157 |
| Sl04-FAM | 32 | 10 | 8.81 | 0.594 | 0.563 | -0.055 | 0.526 | 0.2273 | 0.5295 | 0.445 |
| Sco28-ROX | 34 | 3 | 2.74 | 0.294 | 0.300 | 0.019 | 0.260 | 0.5322 | 0.7354 | 1.000 |
| Mean | 30.78 | 7.33 | 6.86 | 0.616 | 0.607 | -0.013 | 0.570 |  |  |  |
| SD | 2.99 | 3.16 | 2.88 | 0.253 | 0.241 | 0.079 | 0.235 |  |  |  |
