# Supplementary material for: Genetic Diversity and Inter‐Specific Phylogeny of Three Sympatric Cetacean Species (Stenella spp.) in Thai Territorial Waters Based on Mitochondrial and Nuclear DNA Markers
Source: Ecol Evol. 2025 Oct 12;15(10):e72322. doi: 10.1002/ece3.72322 (PMC12516012; doi:10.1002/ece3.72322)
Supplement: Supplementary file 7 — Table S6: The detail of microsatellite loci Stenella longirostris used in this study. [file ECE3-15-e72322-s007.docx]

**The genetic diversity and inter-specific phylogeny of three sympatric cetacean species (*Stenella* spp.) in Thai territorial waters based on mitochondrial and nuclear DNA markers**

Promporn Piboon^1^, Janine Brown^2^, Patcharaporn Kaewmong^3^, Kongkiat Kittiwattanawong^4^ Sarisa Klinhom^1^, Toshiaki Yamamoto^5^, and Korakot Nganvongpanit^1,^*

^1^ The School of Veterinary Medicine, Faculty of Veterinary Medicine, Chiang Mai University, Chiang Mai 50100, Thailand.

^2^ Smithsonian Conservation Biology Institute, Center for Species Survival, 1500 Remount Rd, Front Royal, VA, United States.

^3^ Phuket Marine Biological Center, Phuket 83000, Thailand.

^4^ Department of Marine and Coastal Resources, Ratthaprasasanabhakti Building (Building B) The Government Complex, Bangkok 10210, Thailand

^5^ Department of Veterinary Nursing and Technology, Nippon Veterinary and Life Science University, Musashino, Tokyo, Japan

* Correspondence: korakot.n@cmu.ac.th

E-mail:

PP = promporn.piboon@cmu.ac.th

JB= BrownJan@si.edu

PK = marineanimal.vet@gmail.com

KK = kkongkiat@gmail.com

SK= Yui.sarisarisa@gmail.com

TY= tyamamoto@nvlu.ac.jp

KN = korakot.n@cmu.ac.th

**Table S6.** The detail of microsatellite loci *Stenella longirostris* used in this study

| Locus | N | Na | AR | Ho | He | Fis | PIC | PID | PIDsibs | P(HWE) |
| --- | --- | --- | --- | --- | --- | --- | --- | --- | --- | --- |
| Sl9-69-FAM | 40 | 8 | 7.63 | 0.725 | 0.757 | 0.043 | 0.711 | 0.1002 | 0.4012 | 0.711 |
| Slo4-FAM | 39 | 6 | 5.47 | 0.385 | 0.398 | 0.033 | 0.374 | 0.3879 | 0.6507 | 0.102 |
| Slo9-HEX | 40 | 2 | 2.00 | 0.3 | 0.425 | 0.297 | 0.332 | 0.4246 | 0.6462 | 0.131 |
| EV37-HEX | 39 | 15 | 13.72 | 0.897 | 0.898 | 0.000 | 0.876 | 0.0236 | 0.3128 | 0.461 |
| EV1-TAMRA | 37 | 11 | 10.50 | 0.811 | 0.851 | 0.048 | 0.821 | 0.0441 | 0.3414 | 0.017 |
| Sl1-25-TAMRA | 36 | 12 | 11.55 | 0.778 | 0.822 | 0.055 | 0.790 | 0.0562 | 0.3588 | 0.015 |
| Sco55-ROX | 34 | 6 | 5.69 | 0.294 | 0.341 | 0.138 | 0.322 | 0.4547 | 0.6959 | 0.080 |
| Sl8-49-FAM | 38 | 13 | 12.21 | 0.921 | 0.895 | -0.030 | 0.872 | 0.0250 | 0.3148 | 0.524 |
| Sd8-FAM | 39 | 12 | 11.16 | 0.769 | 0.843 | 0.088 | 0.817 | 0.0435 | 0.3449 | 0.256 |
| Sco28-ROX | 38 | 7 | 6.50 | 0.474 | 0.537 | 0.120 | 0.498 | 0.2533 | 0.5483 | 0.045 |
| EV94-ROX | 29 | 17 | 17.00 | 0.862 | 0.927 | 0.071 | 0.904 | 0.0147 | 0.2983 | 0.543 |
| Sco11-HEX | 37 | 6 | 5.52 | 0.324 | 0.375 | 0.137 | 0.351 | 0.4163 | 0.6691 | 0.024 |
| Slo15-HEX | 34 | 6 | 5.70 | 0.5 | 0.575 | 0.132 | 0.514 | 0.2403 | 0.5270 | 0.711 |
| EV104-HEX | 33 | 4 | 3.88 | 0.455 | 0.474 | 0.042 | 0.420 | 0.3308 | 0.5992 | 0.850 |
| Mean | 36.64 | 8.93 | 8.47 | 0.607 | 0.651 | 0.084 | 0.614 |  |  |  |
| SD | 3.15 | 4.41 | 4.26 | 0.237 | 0.224 | 0.080 | 0.231 |  |  |  |
