# Supplementary material for: Genetic Diversity and Inter‐Specific Phylogeny of Three Sympatric Cetacean Species (Stenella spp.) in Thai Territorial Waters Based on Mitochondrial and Nuclear DNA Markers
Source: Ecol Evol. 2025 Oct 12;15(10):e72322. doi: 10.1002/ece3.72322 (PMC12516012; doi:10.1002/ece3.72322)
Supplement: Supplementary file 8 — Table S7: The detail of most likely K value using a combined dataset (10 loci), Stenella attenuata (8 loci), Stenella coeruleoalba (9 loci) and Stenella longirostris (14 loci) from multiple criteria, including the Pritchard method (Pr[X|K]), the Evanno method (ΔK), and the Parsimony index (PI). [file ECE3-15-e72322-s006.docx]

**The genetic diversity and inter-specific phylogeny of three sympatric cetacean species (*Stenella* spp.) in Thai territorial waters based on mitochondrial and nuclear DNA markers**

Promporn Piboon^1^, Janine Brown^2^, Patcharaporn Kaewmong^3^, Kongkiat Kittiwattanawong^4^ Sarisa Klinhom^1^, Toshiaki Yamamoto^5^, and Korakot Nganvongpanit^1,^*

^1^ The School of Veterinary Medicine, Faculty of Veterinary Medicine, Chiang Mai University, Chiang Mai 50100, Thailand.

^2^ Smithsonian Conservation Biology Institute, Center for Species Survival, 1500 Remount Rd, Front Royal, VA, United States.

^3^ Phuket Marine Biological Center, Phuket 83000, Thailand.

^4^ Department of Marine and Coastal Resources, Ratthaprasasanabhakti Building (Building B) The Government Complex, Bangkok 10210, Thailand

^5^ Department of Veterinary Nursing and Technology, Nippon Veterinary and Life Science University, Musashino, Tokyo, Japan

* Correspondence: korakot.n@cmu.ac.th

E-mail:

PP = promporn.piboon@cmu.ac.th

JB= BrownJan@si.edu

PK = marineanimal.vet@gmail.com

KK = kkongkiat@gmail.com

SK= Yui.sarisarisa@gmail.com

TY= tyamamoto@nvlu.ac.jp

KN = korakot.n@cmu.ac.th

**Table S7.** The detail of most likely K value using a combined dataset (10 loci), *Stenella attenuata* (8 loci)*, Stenella coeruleoalba* (9 loci) *and*  *Stenella longirostris* (14 loci) from multiple criteria, including the Pritchard method (Pr[X|K]), the Evanno method (ΔK), and the Parsimony index (PI).

| **Species** | **K** | **Mean_Ln(D│ K)** | **DeltaK** | **Parsimony** |
| --- | --- | --- | --- | --- |
| Combine 3 species | 1 | -2283.02 | - | 0.5000 |
|  | 2 | -22137.46 | 9.2440 | 0.7684 |
|  | 3 | **-2056.98** | **21.3659** | **0.8272** |
|  | 4 | -2218.80 | 3.8526 | 0.7829 |
|  | 5 | -2218.31 | - | 0.6764 |
| *Stenella attenuata* | 1 | **-318.03** | - | **0.5000** |
|  | 2 | -318.26 | **1.3316** | -0.5881 |
|  | 3 | -319.73 | 0.6454 | -0.5476 |
|  | 4 | -320.18 | 0.8448 | -0.6583 |
|  | 5 | -318.80 | - | -0.6924 |
| *Stenella coeruleoalba* | 1 | **-779.30** | - | **0.5000** |
|  | 2 | -780.22 | 0.1815 | -0.1306 |
|  | 3 | -781.26 | **0.9695** | -0.0041 |
|  | 4 | -781.14 | 0.6905 | 0.1082 |
|  | 5 | -781.80 | - | -0.2334 |
| *Stenella longirostris* | 1 | **-1614.21** | - | **0.5000** |
|  | 2 | -1618.87 | **4.2410** | -0.0833 |
|  | 3 | -1637.64 | 0.5796 | -0.0756 |
|  | 4 | -1644.87 | 2.4539 | -0.0609 |
|  | 5 | -1699.48 | - | -0.0046 |
